# Assessing Cognitive Outcomes in Coma Survivors: A Literature Review

**DOI:** 10.3390/brainsci13010096

**Published:** 2023-01-04

**Authors:** Allison Frantz, Natalia Incio Serra, Aracely Lopez Almendariz, Catherine Duclos, Adrian M. Owen, Stefanie Blain-Moraes

**Affiliations:** 1 Montreal General Hospital, Research Institute of the McGill University Health Center, Montreal, QC H3G 1A4, Canada; 2 Integrated Program in Neuroscience, Faculty of Medicine, McGill University, Montreal, QC H3A 1A1, Canada; 3 School of Physical and Occupational Therapy, Faculty of Medicine, McGill University, Montreal, QC H3G 1Y5, Canada; 4 Department of Anesthesiology and Pain Medicine, Université de Montréal, Montreal, QC H3C 3J7, Canada; 5 Hôpital du Sacré-Coeur de Montréal, Centre Intégré Universitaire de Santé de Services Sociaux du Nord-de-l’île-de-Montréal, Montreal, QC H3C 3J7, Canada; 6 Department of Physiology and Pharmacology and Department of Psychology, University of Western Ontario, London, ON N6A 5C2, Canada

**Keywords:** coma, brain injury, cognitive outcome, cognitive assessment, cognitive instrument, unconsciousness, intensive care, critical care

## Abstract

(1) Background: Although cognitive impairments in coma survivors are common, methods of measuring long-term cognitive outcomes in this population are inconsistent, precluding the development of a strong evidence-base to support clinical decision making. In this literature review, we identify and characterize the measures used to track cognitive recovery in coma survivors to data. (2) Methods: We extracted the instrument used for cognitive assessment, the cognitive domains assessed, methods administration and scoring, and timing of assessment from 134 of 996 screened records. (3) Results: A total of 133 unique cognitive tests and cognitive testing batteries were identified, with 97 cognitive instruments used in less than three articles. The instruments assessed 20 different cognitive domains, with 73 articles also using tests that assess general “cognitive ability”. Cognitive instruments ranged from subjective assessments to comprehensive cognitive batteries. There were inconsistent points of reference for the timing of assessment across studies, with few studies repeating assessments at more than one time point, and arbitrary time intervals between tests. (4) Conclusions: Overall, this review illustrates the enormous disparity between studies that track cognitive outcome in coma survivors, and the need for a systematic, patient-accessible method of assessing cognitive functioning in future studies with this population.

## 1. Introduction

Individuals who are in a coma following a brain injury typically require extensive hospitalization in intensive care units (ICU) to survive their injury and regain their cognitive functions. Although recent large-scale efforts have been launched to improve the treatment and management of coma patients [1], the improvement of their consciousness and cognitive recovery remains hindered by gaps in longitudinal patient assessment, outcome prognostication and treatment. Addressing these gaps is dependent, in part, on the availability of a systematic, patient-accessible method of tracking outcomes in coma survivors. To date, primary outcomes of ICU survivors have focused predominantly on functional outcomes and mortality [2,3]. However, 40–100% of patients requiring care in a ICUs subsequently exhibit cognitive impairment [4,5,6,7,8], regardless of age at injury [9]. Thus, it is critical to identify and standardize a set of long-term cognitive outcomes that are sensitive to the dynamic and complex changes in cognitive ability in coma survivors to provide crucially needed support for clinical decision-making for this population. 

Inconsistencies in measuring long-term cognitive outcomes in coma survivors have been widely acknowledged [10,11]. This heterogeneity can be broadly traced to four sources. First, as recovery from coma involves the return of cognition *and* consciousness, the assessment of cognition is often entangled with the assessment of consciousness. Behavioural measures of consciousness that are used at the bedside (e.g., Glasgow Coma Scale) and in research (e.g., Coma Recovery Scale-Revised [12]) typically associate command-following with the return of consciousness, though command-following can also be considered an indication of basic cognitive functioning. Second, methods of assessment can vary from objectively scored tests of participant responses (e.g., the digit span memory task [13]) to subjective scores generated from a trained observer (e.g., the Functional Independence Measure [14]). Third, although comprehensive cognitive testing batteries exist, these are often too time-consuming to administer at the bedside or to individuals with limited attention. Consequently, clinicians and researcher select a subset of cognitive domains for assessment, ranging from memory, to executive functions, to attention, to language and learning. Fourth, longitudinal studies remain rare, and standards for the timing of cognitive assessment do not currently exist. 

In this article, we review existing literature to systematically identify and characterize trends for measuring cognitive outcomes in coma survivors. Specifically, we aim to identify the measures used to track cognitive recovery in coma survivors to date, and to characterize the frequency and timepoints of these assessments. 

## 2. Materials and Methods

A professional librarian at the McGill University Library assisted with the development of a search strategy and search terms based on our research question and discussions with experts in brain injury and critical care. Three search concepts were identified within the study objectives: “unresponsiveness”, “brain-injured”, and “cognitive outcomes”. As ICU patients with a primary cardiac injury can suffer a secondary brain injury, we targeted studies of brain-injured patients as well as post-cardiac-arrest patients. The search strategy and terms for both targets are shown in Table 1 and Table 2.

The literature search was conducted within the PubMed database in March 2021. We included English-language, primary research articles that measured behavior-based assessments of cognition in adult (>18 years of age) coma survivors. We excluded studies that used neurological proxies to assess cognition (e.g., event-related potentials extracted from an electroencephalogram), articles that did not use validated cognitive tests, and articles whose primary outcomes were functional. We also excluded abstracts, case reports and literature reviews, and articles where participants did not suffer a coma or a period of prolonged unconsciousness. 

**Figure 1:**
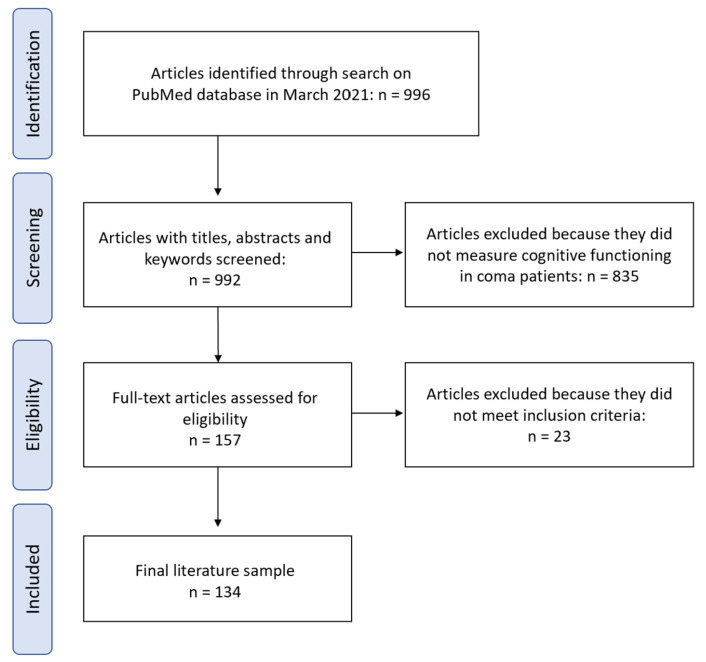
Flowchart outlining the protocol adopted in this narrative review.

All articles were uploaded to Rayyan—an online systematic literature review tool. To verify eligibility, two authors (A.F. and N.I.S.) independently screened the titles and abstracts of articles identified by the search. Discrepancies were resolved by consensus. The same two authors then conducted full-text review and extracted the following data from each study: cognitive instrument(s) administered, timing of cognitive assessment(s), and frequency of assessment(s). Results were grouped by the number of occurrences of each cognitive instrument, the cognitive domain(s) assessed (e.g., memory, attention, processing speed), how the cognitive tests were administered and scored, and the timing of assessment (Figure 1).

## 3. Results

Our search yield 996 records. After removal of 4 duplicates, 835 studies were excluded based on contents of title and abstract. Specifically, the search yielded many studies that referenced “cognition” but that did not measure cognitive functioning in coma patients, leading to a high rate of article exclusion. After full-text review of 157 articles, 23 were excluded, yielding 134 that met the inclusion criteria and were included in this review (Supplementary Table S1).

### 3.1. Cognitive Instruments Administered

The instruments used to assess cognition in coma survivors were extracted from each article, and the total occurrence of each instrument was recorded. A comprehensive list of all cognitive instruments and their total occurrence is presented in Supplementary Table S2. Across 134 articles, a total of 133 unique cognitive tests and cognitive testing batteries were identified. Among these, 97 cognitive instruments were used in less than three articles. Descriptions and method of administration of the 36 cognitive instruments that appeared in three or more articles are presented in Supplementary Table S3. 

**Figure 2:**
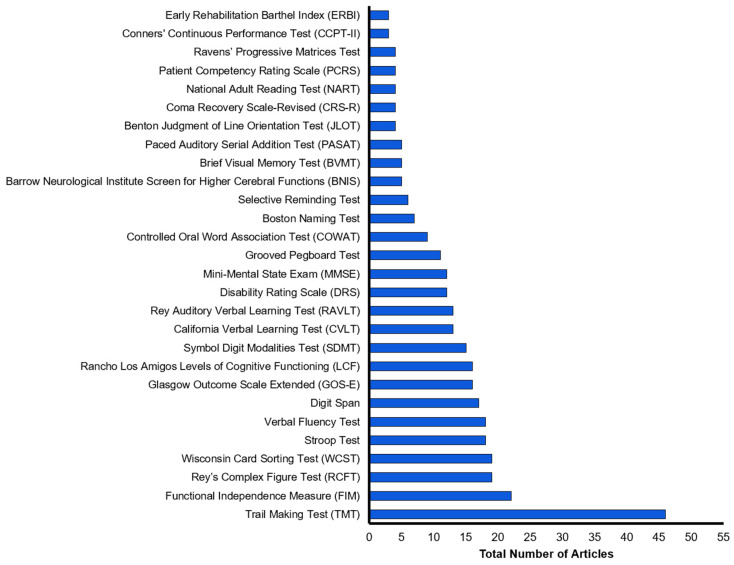
Cognitive instruments using single tests appearing in three or more articles included in this review. Articles are arranged by frequency of appearance.

**Figure 3:**
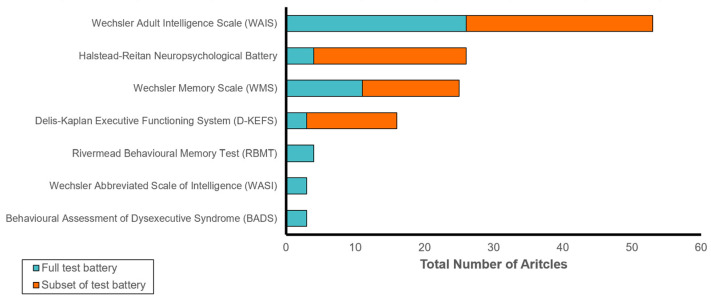
Cognitive instruments using test batteries or battery subsets appearing in three or more articles included in this review. Articles are arranged by frequency of appearance.

The most frequently administered cognitive instrument for coma survivors was the Trail-Making Test (46 articles; 34.3%). Other widely used cognitive instruments included the Functional Independence Measure (22 articles; 16.4%); Rey’s Complex Figure Test (19 articles; 14.2%); the Wisconsin Card Sorting Test (19 articles; 14.2%); the Stroop test (18 articles; 13.4%); the Verbal Fluency Test (18 articles, 13.4%); and the Digit Span test (17 articles; 12.7%). The total occurrence of cognitive instruments using single tests appearing in three or more articles is presented in Figure 2; the total occurrence of cognitive instruments using a test battery, or a subset of a test battery is presented in Figure 3.

A subset of articles used a cognitive testing battery to assess outcomes in coma survivors, rather than an individual instrument. The most frequently used cognitive batteries were the Weschler Adult Intelligence Scale (53 articles; 37.3%), the Halstead–Reitan Neuropsychological Battery (27 articles, 20.1%) and the Weschler Memory Scale (25 articles; 14.9%). However, most articles did not use the full cognitive battery, and focused on a selection of subscales: of the articles that used the Weschler Adult Intelligence Scale, only 23 (43%) used the full battery; of those that used the Weschler Memory Scale, only 11 (44%) used the full battery. A comprehensive list of cognitive testing batteries, their subtests, and their frequency of use is presented in Supplementary Table S3. 

Ten studies (7.5%) did not specify the instruments used to assess cognition. Outcome assessments are described as “standardized neuropsychological examination” [15], “other measures of attention, speed, and memory” [16] or “no standardized set of tests used” [17].

### 3.2. Cognitive Domains Assessed

**Figure 4:**
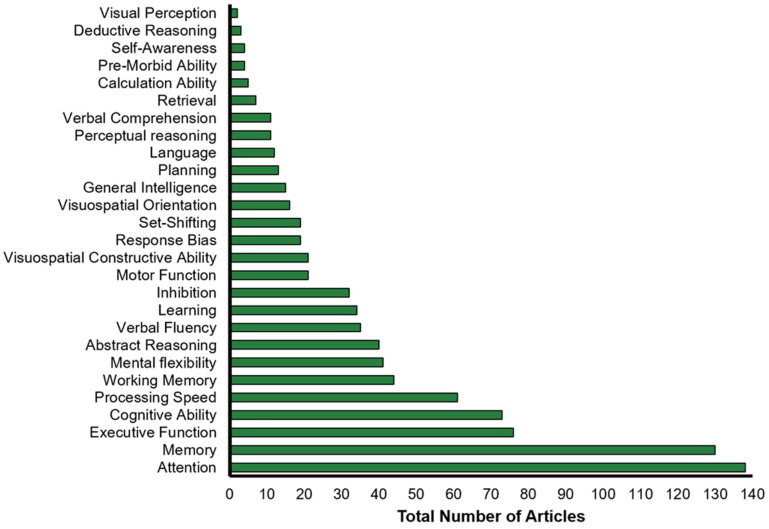
Number of articles that assess each cognitive domain. Only cognitive instruments that were listed in three or more articles are included in this analysis.

We categorized each of the 133 cognitive instruments identified in our review according to the cognitive domain they targeted. The number of tests associated with each cognitive domain, and the total number of articles that used these tests are presented in Table 3. The total number of articles assessing each cognitive domain for cognitive instruments reported in more than three articles is presented in Figure 4. The most frequently assessed cognitive domains were attention (10 tests, 138 occurrences), memory (11 tests, 130 occurrences) and executive function (5 tests, 76 occurrences). Many articles also used tests of general “cognitive ability” (6 tests, 73 occurrences), without further breakdown into specific cognitive domains.

### 3.3. Method of Administration

We also categorized the 133 cognitive instruments used to assess outcome in coma survivors based upon the method of administration and scoring (Table 4). The cognitive instruments that were used most frequently across articles were supervised by an examiner upon administration and scored using a rater’s manual, a quantitative measure of performance (e.g., time to completion), or automatically on a computer. In contrast, instruments that measured “general cognitive ability” were administered by an examiner who interviewed or observed the participant and scored based on the examiner’s subjective assessment. 

### 3.4. Timing of Cognitive Assessments 

The timing of cognitive outcome assessment was extracted from each article, specifically, the reference point relative to milestones in injury and recovery, and the total number of assessments and the interval between assessments. 

“Time since injury” was the most common reference point (74 articles; 55%). The number and interval of assessments relative to this reference point are presented in Figure 3. Eighteen (13.4%) articles used “time since admission to rehabilitation” as a reference point. The remaining articles used one of four other reference points: “time since discharge from rehabilitation”, “time since admission to intensive care”, “time since discharge from intensive care”, and “time since intervention/treatment”. The total number of assessments and testing intervals relative to these other reference points are presented in Figure 4. Ten articles (7.5%) did not specify the timing of cognitive outcome assessments. 

**Figure 5:**
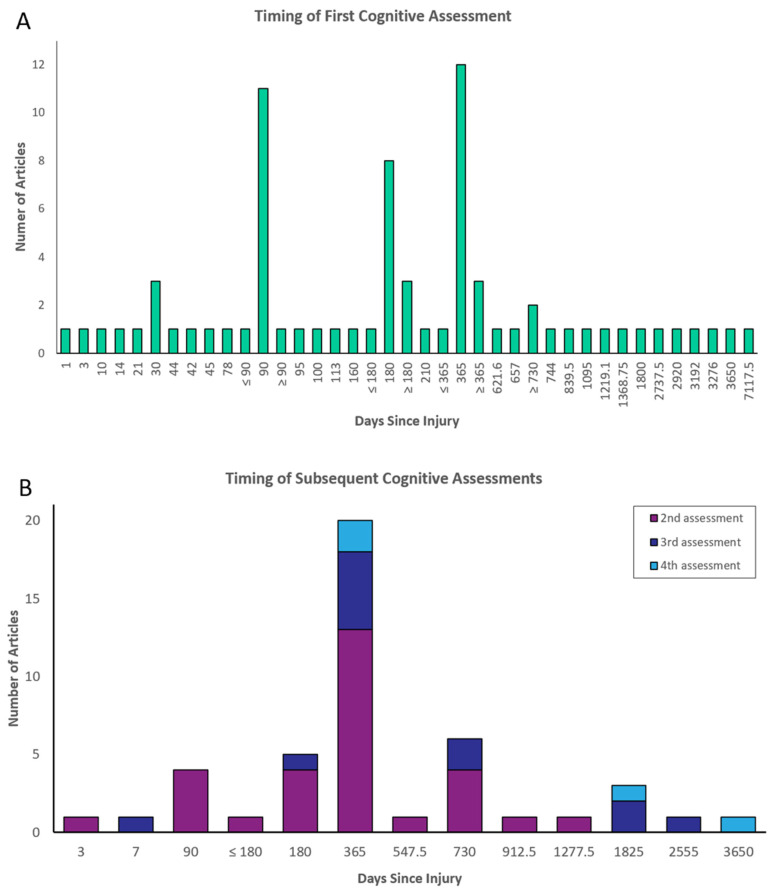
Number and interval of assessments relative to time since injury. (**A**). First cognitive assessment timepoint. (**B**). Timing of second, third and fourth cognitive assessment within a single study.

**Figure 6:**
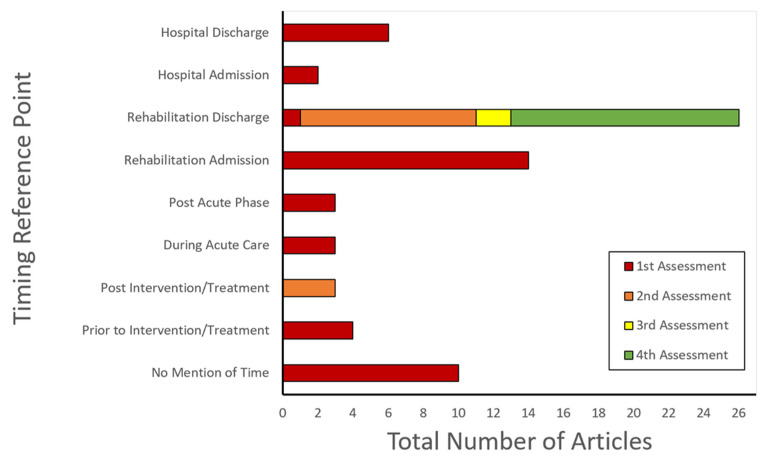
Total number of articles assessing cognitive outcomes in coma survivors relative to reference points other than “time since injury”. Red bars indicate a first assessment timepoint, orange bars indicate a second assessment, yellow bars indicate a third assessment, and green bars indicate a fourth assessment within the same study.

Figure 5 and Figure 6 also show the total number of assessments conducted in each study. Across all articles, 74 conducted a minimum of one cognitive outcome assessment; 30 conducted a minimum of two assessments; 12 conducted a minimum of three assessments; and four articles conducted four assessments. 

Among all studies, the most common time-points for assessing cognition were at 3 months (15 articles; 11 first assessment, 4 second assessment), 6 months (13 articles; 8 first assessment, 4 second assessment, 1 third assessment), and one-year post-injury (31 articles; 11 first assessment, 13 second assessment, 5 third assessment, 2 fourth assessment). No articles assessed cognitive outcome in coma survivors at more than 4 time-points.

## 4. Discussion

The prevalence of coma survivors is steadily increasing as a result of a growing incidence of critical illness [18] and advances in medical technologies that reduce ICU mortality. A recent systematic review of ICU survivors suggested that cognitive impairment in this population is common, ranging between 35% to 81% at 3 months after ICU discharge [11]. Given that cognitive function is a strong predictor of quality of life, these trends constitute an increasing public health problem that needs to be matched with increased awareness and treatment [19].

Our review identified 134 articles that assessed cognitive ability in coma survivors, either as characterization of the natural history of ICU-related cognitive impairment, or as a measure of intervention effectiveness. Most studies did not include cognition as a primary outcome: cognitive outcomes were typically assessed as secondary endpoints, or in response to an intervention. Collectively, our results illustrate the enormous heterogeneity in the type of cognitive instrument, the cognitive domains assessed, the method of test administration and the timing of assessment across studies. Across the 134 articles, 133 unique cognitive instruments were used to assess outcome; only 36 of these appeared in three or more articles. The cognitive measures targeted 20 different cognitive domains. Although attention, memory, executive function and general cognitive ability were more commonly targeted, none was dominant across articles. The timing of assessment was heterogeneous both with respect to the reference point used in each study, and with respect to the number and interval of assessments. Altogether, our results illuminate the paucity of a systematic and consistent approach to measuring cognitive outcomes in coma survivors, which is required to providing critically needed support for clinical decision-making.

This review did not identify many studies that deployed a comprehensive cognitive battery that measured cognition at more than one time point. Such measures have historically been difficult to gather due to lack of comprehensive, easy-to-administer neurocognitive tests. Currently, assessment of cognitive function requires that patients attend a clinic where specially trained personnel administer standard cognitive batteries. This model has several limitations including the length of these testing sessions, patient inconvenience of travelling to clinic assessments, high costs associated with employing trained personnel, and high rates of patient no-shows. Furthermore, there are increased challenges in following patients who undertake a complex rehabilitation trajectory and go through several institutions. The impracticality of their use in routine clinical settings may explain the enormous variability present in our review, as each study site is pragmatically constrained to a self-selected subset of cognition instruments, deployed on a minimal number of occasions. Some tests are better adapted to the acute phase, while others are more suited for the chronic phase. These phases are generally associated with different levels of cognitive dysfunction and may require tests that are adapted to these settings. For example, in the acute phase level of consciousness and orientation are the focus of most tests. Once patients regain full consciousness and are orientated, more granular cognitive testing is more appropriate. Comprehensive web-based batteries (e.g., Cambridge Brain Sciences [9,20]) may be a viable alternative to traditional cognitive assessment, presenting a systematic, patient-accessible method of tracking cognitive recovery in coma survivors.

Over half of the articles identified in this review deployed measures of “general cognitive ability”. These measures—which include the Functional Independence Measure, the Glasgow Outcome Scale, and the Ranchos Los Amigos Levels of Cognitive Functioning—need to be considered separately from the other measures of cognitive outcome included in this review. Assessments of general cognitive ability were performed by an examiner providing a score based on their subjective assessments of a patient’s behavior, rather than on performance-based objective measures. A recent systematic review of cognition in ICU survivors by Honarmand et al. demonstrated that early after discharge, the prevalence of cognitive impairment appeared to be higher when objective rather than subjective measures of cognition were used [11]. It is important to note that brain injury survivors tend to underestimate their deficits; the more severe the injury, the more they lack the critical thinking skills to perceive and report their deficits. Although the subjective measures in Honarmand’s review refer to patient-reported outcomes and are thus not directly comparable to third-party subjective assessments, their results nonetheless indicate a potential non-equivalence between the two methods of administration. Both types of cognitive assessment are useful for tracking cognitive recovery in coma survivors: subjective cognitive assessments are typically used in the acute stages of recovery, when patients are unable to operate a computer, or are otherwise functionally unable to perform an objective cognitive test. Furthermore, the tests used in the acute stage assess different functions than those tailored for the chronic phase; rather than assessing high-level cognitive functions in specific cognitive domains, those tests used in the acute phase focus on assessing the burden of residual cognitive deficits on everyday function. In future research, a deliberate selection of subjective and objective cognitive measurements in response to patient recovery may be optimal for sensitively tracking the dynamic and complex changes in cognitive ability in coma survivors. 

The results of our review need to be considered in light of a number of limitations. First, we only included behavioral measures of cognition within the scope of this review. Event-related brain responses, such as event-related potentials (ERPs) have shown strong potential for detecting cognitive ability in the absence of behavioral responsiveness [21], such as in coma. Similarly, command-following detection through neuroimaging modalities such as functional magnetic resonance imaging (fMRI) have been used to indicate consciousness in behaviorally unresponsive individuals, but can also be considered indications of underlying cognitive ability [22]. Although promising, these brain imaging-based measures of cognitive ability have mostly been confined to the realm of research studies, with minimal deployment in clinical or outpatient settings. As they are broadly inaccessible, they remain optimal for custom patient assessments, rather than as a generalized measure of cognitive outcome in coma survivors. Second, our review included studies that assessed cognition as part of a natural trajectory of coma recovery, as well as studies that assessed cognition in response to an intervention after surviving a coma. As the interventions may have been tailored for a particular cognitive domain, this may have biased the choice of instruments for the study. Third, our review was only conducted in the PubMed database, and thus may not represent a complete and comprehensive overview of all cognitive outcomes used to assess coma survivors. However, as the objective of this review was to highlight trends rather than to conduct a meta-analysis of the literature, this limitation has minimal influence on the results. Finally, our results relied on the description of the cognitive assessments and instruments provided by the study authors. Different versions of some cognitive instruments exist; if the study authors did not provide complete details about the instrument they used, we could not determine if they could be grouped with other studies. 

## 5. Conclusions

Although cognitive impairments in coma survivors are common, our literature review revealed that there are no trends across published studies with respect to cognitive instruments, cognitive domain, methods of assessment or timing of assessments used to measure cognitive outcomes. The development of effective preventative, therapeutic and rehabilitation interventions for coma survivors is dependent on a consistent and systematic method of tracking cognitive outcomes in this population. This review article highlights the need for a patient-accessible cognitive battery that can be used repeatedly and easily by patients who survive coma, enabling the development of evidence-based treatments for the growing public health problem of ICU-related cognitive impairment. 

## Figures and Tables

**Table 1:** Search strategy for cognitive outcomes of patients with a primary brain injury (Concept 1) following a period of unconsciousness. All terms in the table were included in the search. Concepts are separated into columns where each term in a column was separated by “OR” in the search and each column (concept) was separated by “AND”.

Concept 1: Brain INJURY	Concept 2: Coma	Concept 3: Cognition
“brain injuries, traumatic” [MeSH Terms]	“coma” [MeSH Terms]	“cognition” [MeSH Terms]
“craniocerebral trauma” [MeSH Terms]	“coma, post head injury” [MeSH Terms]	“cognitive disorders” [MeSH Terms]
“severe traumatic brain injur *” [Title/Abstract]	“unconsciousness” [MeSH Terms]	“cognitive dysfunction” [MeSH Terms]
NOT “concussion”	“unresponsiveness” [Title/Abstract]	“cognitive dysfunction” [MeSH Terms]
NOT “mild traumatic brain injury”	“coma” [Title/Abstract]	“cognitive impairment” [Title/Abstract]
	“non-responsive” [Title/Abstract]	“cognitive recovery” [Title/Abstract]
		“cognitive deficit” [Title/Abstract]

**Table 2:** Search strategy for cognitive outcomes of patients with a brain injury secondary to cardiac arrest (Concept 1) following a period of unconsciousness. All terms in the table were included in the search. Concepts are separated into columns where each term in the column was separated by “OR” in the search and concepts were separated by “AND”.

Concept 1: Cardiac Arrest	Concept 2: Coma	Concept 3: Cognition
“heart arrest” [MeSH Terms]	“coma” [MeSH Terms]	“cognition” [MeSH Terms]
“myocardial infarction” [MeSH Terms]	“coma, post head injury” [MeSH Terms]	“cognitive disorders” [MeSH Terms]
“cardiac arrest” [Title/Abstract]	“unconsciousness” [MeSH Terms]	“neurocognitive disorders” [MeSH Terms]
“myocardial infarction” [Title/Abstract]	“unresponsiveness” [Title/Abstract]	“cognitive dysfunction” [MeSH Terms]
	“coma” [Title/Abstract]	“cognitive impairment” [Title/Abstract]
	“non-responsive” [Title/Abstract]	“cognitive recovery” [Title/Abstract]
		“cognitive deficit” [Title/Abstract]

**Table 3:** **Cognitive domains targeted in outcome assessments of coma survivors.** Cognitive domains are listed alphabetically, along with the number of unique tests used to assess the domain. Tests that appeared in three or more articles are included, along with the total number of articles that included them.

Domains	Tests	No. of Articles
Abstract Reasoning	Category Test from HRNB	11
	Proverb Test from D-KEFS	3
	Raven’s Progressive Matrices	4
	Twenty Questions Subtest from D-KEFS	3
	Wisconsin Card Sorting Test	19
Attention	Conner’s Continuous Performance Test	3
	Grooved Pegboard Test	11
	Mini-Mental State Exam	12
	Rey Auditory Verbal Learning Test	13
	Rhythm Test from HRNB	7
	Sorting Test from D-KEFS	3
	Speech Sounds Perception Test from HRNB	6
	Symbol Digit Modalities Test	18
	Trail Making Test	46
	Wisconsin Card Sorting Test	19
Deductive Reasoning	Word Context Test from D-KEFS	3
Executive function	Conner’s Continuous Performance Test	3
	D-KEFS	3
	Grooved Pegboard Test	11
	Rey Auditory Verbal Learning Test	13
	Trail Making Test	46
General Intelligence	Raven’s Progressive Matrices	4
	Wechsler Adult Intelligence Scale-Revised	11
Inhibition	Color-Word Interference Test from D-KEFS	7
	Stroop Test	18
	Tower Test from D-KEFS	7
Learning	California Verbal Learning Test	13
	Rey Auditory Verbal Learning Test	13
	Selective Reminding Test	8
Memory	Benton Visual Retention Test	2
	Brief Visual Memory Test	5
	California Verbal Learning Test	13
	Digit Span	45
	Mini-Mental State Exam	12
	Rey Auditory Verbal Learning Test	13
	Rey’s Complex Figure Test	19
	Rivermead Behavioral Memory Test	4
	Selective Reminding Test	8
	Tactual Performance Test from HRNB	8
	Wechsler Memory Scale	11
Mental Flexibility	Design fluency from D-KEFS	3
	Paced Auditory Serial Addition Test	5
	Trail Making Test from D-KEFS	11
	Wisconsin Card Sorting Test	19
	Word Context Test from D-KEFS	3
Motor Function	Finger tapping Test from HRNB	10
	Grooved Pegboard Test	11
Perceptual Reasoning	Wechsler Adult Intelligence Test-Revised from D-KEFS	11
Planning	Design Fluency from D-KEFS	3
	Tower Test from D-KEFS	7
	Zoo Map Test from BADS	3
Processing Speed	Paced Auditory Serial Addition Test	5
	Rey’s Complex Figure Test	19
	Symbol Digit Modalities Test	18
	Trail Making Test from HRNB	8
	Wechsler Adult Intelligence Scale-Revised	11
Response Bias	Rey’s Complex Figure Test	19
Retrieval	Boston Naming Test	7
Self-Awareness	Patient competency Rating Scale	4
Set-Shifting	Wisconsin Card Sorting Test	19
Working Memory	Sorting Test from D-KEFS	3
	Wechsler Adult Intelligence Scale-Revised	11
	Wechsler Memory Scale	11
	Wisconsin Card Sorting Test	19
Language	Mini-Mental State Exam	12
Verbal Comprehension	Wechsler Adult Intelligence Scale-Revised	11

D-KEFS: Delis–Kaplan Executive Functioning System; HRNB: Halstead–Reitan Neuropsychological Battery.

**Table 4:** Methods of administering and scoring assessments of cognition in coma survivors.

Method of Administration	Test
Questionnaire completed by trained rater	Mini-Mental State Exam
Trained examiner provides a score based on participant’s abilities (gathered through interview or observation)	Coma Recovery Scale—Revised, Functional Intelligence Measure, Glasgow Outcome Scale—Extended, Ranchos Levels of Cognitive Functioning, Early Rehabilitation Barthel Index
Test supervised by examiner, score based on time for performance	Color Trails Test, Grooved Pegboard, Trail Making Test, Delis–Kaplan Executive Functioning System, Halstead–Reitan Neuropsychological Battery (Trail Making Test, Tactual Performance Test), Weschler Adult Intelligence Scale (Block Design)
Test completed on computer that generates score automatically	Conners’ Continuous Performance Test
Test supervised by examiner, score based on performance and instructions from rater’s manual	Behavioral Assessment of Dysexecutive Syndrome, Judgment of Line Orientation Test, Boston Naming Test, Brief Visual Memory Test, California Verbal Learning Test, Controlled Oral Word Associate Test, Digit Span, Delis–Kaplan Executive Functioning System (Verbal Fluency, Design Fluency, Color-Word Interference, Sorting Test, 20Q, Tower Test), Halstead–Reitan Neuropsychological Battery (Category Test, Finger Tapping Test, Rhythm Test, Speech Sound Perception Test, Sensory Perceptual Test, Lateral Dominance), National Adult Reading Test, Paced Auditory Serial Addition Test, Raven’s Progressive Matrices, Rivermead Behavioral Memory Test, Rey Auditory Verbal Learning Test, Rey’s Complex Figure Test, Selective Reminding Test, Stroop Test, Verbal Fluency Test, Weschler Adult Intelligence Scale (Similarities, Matrix Reasoning, Digit Span, Letter-Number Sequencing, Picture Completion, Arithmetic, Digit Symbol, Symbol Search), Wisconsin Card Sorting Test, Weschler Memory Scale (Spatial Addition, Design Memory, Symbol Span, Logical Memory, Verbal Paired Associates, Visual Reproduction)

## Data Availability

Not applicable.

## Supplementary Materials

The following supporting information can be downloaded at: https://www.mdpi.com/article/10.3390/brainsci13010096/s1, Table S1: Final literature sample; Table S2: Total occurrence of cognitive instruments applied to coma survivors; Table S3: Description and method of administration of cognitive instruments used in three or more articles.
