# Supplementary material for: Assessing Cognitive Outcomes in Coma Survivors: A Literature Review
Source: Brain Sci. 2023 Jan 4;13(1):96. doi: 10.3390/brainsci13010096 (PMC9856711; doi:10.3390/brainsci13010096)
Supplement: Supplementary file 1 [file brainsci-13-00096-s001.zip › brainsci-2085947-supplementary.pdf]

Online Data Supplement

Supplementary Table S1: Final literature sample

Authors	Title	Tests or batteries used	Timing of assessments
Aharon-Peretz J and Kliot D and Amyel-Zvi E and Tomer R and Rakier A and Feinsod M	Neurobehavioral consequences of closed head injury in the elderly.	Digit span; List of 8 unrelated words; visual reproduction, Weschler Memory Scale; Supermarket list; WAIS-R (similarities subtest)	At discharge and 6 weeks later
Alexander MP and Lafleche G and Schnyer D and Lim C and Verfaellie M	Cognitive and functional outcome after out of hospital cardiac arrest.	RAVLT; BVMT-R; TMT A&B; WCST; Verbal fluency; BNT (alternate items); Peabody picture vocabulary; Judgment of line orientation; Number location; Visual discrimination; Grooved pegboard; Finger tapping	Time to testing mean: 113 days (time since injury)
Alosco ML and Tripodis Y and Baucom ZH and Mez J and Stein TD and Martin B and Haller O and Conneely S and McClean M and Nosheny R and Mackin S and McKee AC and Weiner MW and Stern RA	Late contributions of repetitive head impacts and TBI to depression symptoms and cognition.	CogState Brief Battery (CBB)30; Lumos Labs NeuroCognitive Performance Test	Every 6 months
Ariza M and Mataro M and Poca MA and JunqueC and Garnacho A and Amoros S and Sahuquillo J	Influence of extraneurological insults on ventricular enlargement and neuropsychological functioning after moderate and severe traumatic brain injury.	Selective Reminding Test; Rey-Osterrieth Complex Figure; TMT A&B; COWAT; Grooved pegboard test; SDMT	6 months post-injury
Avesani R and Carraro E and Armani G and Masiero S	Exploring variables associated with rehabilitation length of stay in brain injuries patients.	Disability Rating Scale (DRS); FIM; LCF	At admission and discharge from rehabilitation center
Bagnato S and Boccagni C and Prestandrea C and Sant'Angelo A and Castiglione A and Galardi G	Prognostic value of standard EEG in traumatic and non-traumatic disorders of consciousness following coma.	LCF	At admission and 3 months post admission to rehabilitation center.

Bangirana P and Giordani B and Kobusingye O and Murungyi L and Mock C and John CC and Idro R	Patterns of traumatic brain injury and six-month neuropsychological outcomes in Uganda.	Cogstate computer-administered neuropsychological test battery	At admission and 6 months post discharge from hospital
Berger-Estilita J and Granja C and Goncalves H and Dias CC and Aragao I and Costa-Pereira A and Orwelius L	A new global health outcome score after trauma (GHOST) for disability, cognitive impairment, and health-related quality of life: data from a prospective cross-sectional observational study.	TMT A&B; COWAT; Rey Auditory Verbal Learning Test (RAVLT); Digit Span Test; Digit Symbol Substitution Test	6 months after discharge
Birle C and Slavoaca D and Muresanu I and Chira D and Vacaras V and Stan AD and Dina C and Strilciuc S	The Effect of Cerebrolysin on the Predictive Value of Baseline Prognostic Risk Score in Moderate and Severe Traumatic Brain Injury.	MMSE; WAIS (Processing Speed Index); Stroop Colour Word Test-Victoria Version	At 10,30 and 90 days post treatment
Bivona U and Costa A and Contrada M and Silvestro D and Azicnuda E and Aloisi M and Catania G and Ciurli P and Guariglia C and Caltagirone C and Formisano R and Prigatano GP	Depression, apathy and impaired self-awareness following severe traumatic brain injury: a preliminary investigation.	LCF; DRS; GOS-E; WCST	At admission
Bondanelli M and Ambrosio MR and Cavazzini L and Bertocchi A and Zatelli MC and Carli A and Valle D and Basaglia N and Uberti EC	Anterior pituitary function may predict functional and cognitive outcome in patients with traumatic brain injury undergoing rehabilitation.	LCF; DRS; FIM	At admission and discharge from in-patient rehabilitation
Bondanelli M and Ambrosio MR and Margutti A and Boldrini P and Basaglia N and Franceschetti P and Zatelli MC and Degli Uberti EC	Evidence for integrity of the growth hormone/insulin-like growth factor-1 axis in patients with severe head trauma during rehabilitation.	LCF; FIM	At least one month after discharge from ICU
Brooke MM and Questad KA and Patterson DR and Valois TA	Driving evaluation after traumatic brain injury.	TMT; Tactual Performance Test; WMS; WAIS-R	3-6 months post injury

Brooks DN and Aughton ME and Bond MR and Jones P and Rizvi S	Cognitive sequelae in relationship to early indices of severity of brain damage after severe blunt head injury.	Raven's progressive matrices; Mill Hill vocabulary scale; Logical memory (WMS); Associate learning (Inglis Paired Associate Test); Rey picture (immediate and delayed); Word fluency tests (WF1 2 and 3); Block design (WAIS)	Once, within two years post-injury
Chabok SY and Kapourchali SR and Leili EK and Saberi A and Mohtasham-Amiri Z	Effective factors on linguistic disorder during acute phase following traumatic brain injury in adults.	Persian Aphasia Test (PAT); Persian version of Mini-Mental State Examination (MMSE)	At discharge (average hospitalization of 1 week after injury).
Chen SH and Thomas JD and Glueckauf RL and Bracy OL	The effectiveness of computer-assisted cognitive rehabilitation for persons with traumatic brain injury.	WAIS-R; WMS (some subsets); Other: category test, TMT A&B form HRNB, WCST, digit span (has forward and backward as stand alone and one subtest of WAIS), logical memory, visual reproduction (immediate and delayed), paired associates (immediate and delayed)	Post-discharge (early part of post-acute rehabilitation). Range of time between testing points varied.
Chiou KS and Sandry J and Chiaravalloti ND	Cognitive contributions to differences in learning after moderate to severe traumatic brain injury.	Selective Reminding Test (SRT); Wechsler Adult Intelligence Scale (WAIS–III); Digit Span Forward & backward (WAIS-III); Symbol Digit Modality Test (SDMT); Delis–Kaplan Executive Functioning System (DKEFS); Paced Auditory Serial Addition Test (PASAT); Wechsler Adult Scales of Intelligence (WASI)	Mean time of assessment since injury for TBI learner = 106.4months (SD=88.2), TBI-non learners=109.2months (SD=117.9)
Cifu DX and Kreutzer JS and Marwitz JH and Rosenthal M and Englander J and High W	Functional outcomes of older adults with traumatic brain injury: a prospective, multicenter analysis.	DRS, LFCS (Rancho), FIM	Within 72 hours of inpatient rehab admission and discharge
Cowen TD and Meythaler JM and DeVivo MJ and Ivie CS 3rd and Lebow J and Novack TA	Influence of early variables in traumatic brain injury on functional independence measure scores and rehabilitation length of stay and charges.	FIM	*Study performed retrospectively* At admission and discharge from rehabilitation
Davis AE and Gimenez A	Cognitive-behavioral recovery in comatose patients following auditory sensory stimulation.	Ranchos Los Amigos Cognitive Functioning (LCF); DRS	Evaluated at the beginning of study (average time of entry to study was 9.4 days after injury) and at completion

de Guise E and Leblanc J and Feyz M and Lamoureux J	Prediction of the level of cognitive functional independence in acute care following traumatic brain injury.	FIM (cognitive & communication)	At discharge from acute care (48 hours to 45 days post-admission)
DE Souza RL and Thais ME and Cavallazzi G and Paim Diaz A and Schwarzbold ML and Nau AL and Rodrigues GM and Souza DS and Hohl A and Walz R	Side of pupillary mydriasis predicts the cognitive prognosis in patients with severe traumatic brain injury.	Letters and Category Fluency<br>Rey Auditory Verbal Learning Test (RAVLT)<br>WMS-III: subtests Logical Memory First Recall (LM 1st), Logical Memory I (LM I), Logical Memory II (LM II) and Visual Reproduction I (VR I), Visual Reproduction II (VR II), Visual Reproduction Recognition (VR Rec)<br>Wechsler Adult Intelligence Scale III (WAIS-III): subtests Digit Span, Similarities, Vocabulary and Block Design.	Just one assessment on average 2.5 (SEM ± 0.2) years after hospitalization(hospital discharge) (range between 1 to 6 years).
de Vos R and de Haes HC and Koster RW and de Haan RJ	Quality of survival after cardiopulmonary resuscitation.	MMSE	Follow-up was at least 3 months after discharge from hospital (varied by patient)
Di Paola M and Phillips O and Costa A and Ciurli P and Bivona U and Catani S and Formisano R and Caltagirone C and Carlesimo GA	Selective Cognitive Dysfunction Is Related to a Specific Pattern of Cerebral Damage in Persons With Severe Traumatic Brain Injury.	Digit span & Digit Span Backward test;<br>15-Word Immediate a Delayed Recall and Prose Immediate and Delayed Recall tests;<br>Modified Card Sorting test<br>Trail Making test (A&B),<br>Tower of London test,<br>Phonological Verbal Fluency test;<br>Attentive Matrices test<br>Raven's Coloured Progressive Matrices–PM47	>2 months since coma (days since injury for dys-episodic group: 183.25, SD 102.91; dys-executive group: 444.43, SD 339.64)
Dikmen S and McLean A Jr and Temkin NR and Wyler AR	Neuropsychologic outcome at one-month postinjury.	Halstead-Reitan neuropsychological test battery and other measures of attention, speed, and memory	As close to 1-month post-injury as possible

Drudge OW and Williams JM and Kessler M and Gomes FB	Recovery from severe closed head injuries: repeat testings with the Halstead-Reitan Neuropsychological Battery.	Halstead-Reitan<br>WAIS	Two timepoints:<br>1) as soon as patient was deemed testable (mean time between injury and testing: 2.6 months)<br>2) 1 year postrauma (mean: 11.5 months)
Dye OA and Saxon SA and Milby JB	Long-term neuropsychological deficits after traumatic head injury with comatosis.	Category test<br>TPT (tactual performance)<br>Speech perception test<br>Rhythm test<br>Finger tapping test<br>TMT A&B<br>WAIS	Half of participants had between 0-18 months between time they awoke from coma and time of testing, half of participants had 18-36 months
Dywan J and Segalowitz SJ and Henderson D and Jacoby L	Memory for source after traumatic brain injury.	Fame judgment task; WCST; BFRT (Benton Facial Recognition Test); CVLT	Time since trauma ranged from 1 to 15 years
Esbjornsson E and Skoglund T and Sunnerhagen KS	Fatigue, psychosocial adaptation and quality of life one year after traumatic brain injury and suspected traumatic axonal injury; evaluations of patients and relatives: a pilot study.	Daily Fatigue Impact Scale (D-FIS)<br>Barrow Neurological Institute Screen for Higher Cerebral Functions (BNIS),<br>European Questionnaire 5 Dimensions health-related quality of life<br>Glasgow Coma Outcome Scale Extended,<br>European Brain Injury Questionnaire (EBIQ) (patient and relative).	The study population was examined in the acute phase, at 6 and 12 months (post TBI)
Failla MD and Myrga JM and Ricker JH and Dixon CE and Conley YP and Wagner AK	Posttraumatic Brain Injury Cognitive Performance Is Moderated by Variation Within ANKK1 and DRD2 Genes.	GOS-E; TMT A&B; Digit Span, WAIS-R subtest, Rey-Osterreith Complex Figure Test; CVLT-II; COWAT; DKEFS Verbal Fluency; Stroop Task	6 and 12 months post TBI

Finnanger TG and Olsen A and Skandsen T and Lydersen S and Vik A and Evensen KA and Catroppa C and H√•berg AK and Andersson S and Indredavik MS	Life after Adolescent and Adult Moderate and Severe Traumatic Brain Injury: Self-Reported Executive, Emotional, and Behavioural Function 2–5 Years after Injury	BRIEF-A; Adult Self-Report (ASR); GCS; GOSE; Grooved Pegboard; D-KEFS; TMT 2-4 from D-KEFS; Color-Word Interference Test Condition (CWIT); SDMT; Conners' Continuous Performance Test II (CPT-II); Continuous, Visual Memory Test (CVMT); Rey-Osterrieth Complex Figure Test; CVLT-II; WCST; Verbal Fluency Test (D-KEFS); Tower Test (D-KEFS)	At 3 months, 12 months and then 2.5 - 5 years post injury
Finnanger TG and Skandsen T and Andersson S and Lydersen S and Vik A and Indredavik M	Differentiated patterns of cognitive impairment 12 months after severe and moderate traumatic brain injury.	WAIS	Patients were tested at both 3 and 12 months postinjury.
Fordyce DJ and Roueche JR and Prigatano GP	Enhanced emotional reactions in chronic head trauma patients.	MMPI; Katz adjustment scale "Standardized neuropsychological examination"	2 groups:<br>Acute group was examined 6 months or less following injury<br>Chronic group was examined over 6 months after injury
Formisano R and Carlesimo GA and Sabbadini M and Loasses A and Penta F and Vinicola V and Caltagirone C	Clinical predictors and neuropsychological outcome in severe traumatic brain injury patients.	Verbal episodic memory (word-list learning, prose recall); Visual episodic memory (Rey figure delayed recall); Word fluency (phonological and categorical); General intelligence (Raven's progressive matrices')	Interval between injury and neuropsychological assessment varied (4-29 months post injury)
Frazzitta G and Zivi I and Valsecchi R and Bonini S and Maffia S and Molatore K and Sebastianelli L and Zarucchi A and Matteri D and Ercoli G and Maestri R and Saltuari L	Effectiveness of a Very Early Stepping Verticalization Protocol in Severe Acquired Brain Injured Patients: A Randomized Pilot Study in ICU.	GCS; CRS-R; DRS; LCF	Third day from the injury (T0), at ICU discharge<br>(T1) and at Rehabilitation discharge (T2)
Fugate JE and Moore SA and Knopman DS and Claassen DO and Wijdicks EF and White RD and Rabinstein AA	Cognitive outcomes of patients undergoing therapeutic hypothermia after cardiac arrest.	TICS (Telephone Interview for Cognitive Status)	Median: 19.5 months post-injury
Furlonger AJ and Sleigh JW and Havill JH and Marsh NV and Kersel DA	Cognitive and psychosocial outcome in survivors of severe traumatic brain injury: correlations with cerebral	SSEP (somatosensory evoked potentials)<br>COWAT (controlled oral word association)	Neuropsych assessments conducted 1 year following injury

	perfusion pressure, frontal lobe damage and somatosensory evoked potentials.		
Gansler DA and Covall S and McGrath N and Oscar-Berman M	Measures of prefrontal dysfunction after closed head injury.	DR (delayed response), DA (delayed alternation), OA (object alternation): from Wisconsin General Test Apparatus<br>WCST; WAIS-R	Time since injury varied: 6-280 months (mean 48). All subjects received neuropsychological testing "post-acutely"
Geffen GM and Encel JS and Forrester GM	Stages of recovery during post-traumatic amnesia and subsequent everyday memory deficits.	RBMT (Rivermead Behavioural Memory Test)	One month after emergence from PTA
Golden Z and Golden CJ	Impact of brain injury severity on personality dysfunction.	WAIS-R; HRNB (Tactual performance test, Speech sounds performance test, Rhythm test, Finger oscillation test, TMT B, DSMT, Category test,<br>Block design); MMPI (Minnesota Multiphasic Personality Inventory)	At least one year post injury
Grubb NR and Simpson C and Sherwood RA and Abraha HD and Cobbe SM and O'Carroll RE and Deary I and Fox KA	Prediction of cognitive dysfunction after resuscitation from out-of-hospital cardiac arrest using serum neuron-specific enolase and protein S-100.	RBMT; Digit span (from revised WMS); NART	Within 24 hours of planned discharge (from hospital)
Hammond FM and Giacino JT and Nakase Richardson R and Sherer M and Zafonte RD and Whyte J and Arciniegas DB and Tang X	Disorders of Consciousness due to Traumatic Brain Injury: Functional Status Ten Years Post-Injury.	FIM	At 1, 2, 5, and 10 years<br>post-injury by telephone
Hart T and Kozlowski AJ and Whyte J and Poulsen I and Kristensen K and Nordenbo A and Heinemann AW	Functional recovery after severe traumatic brain injury: an individual growth curve approach.	FIM	At admission to rehab, discharge, and every 2 weeks in between
Haslam C and Batchelor J and Fearnside MR and Haslam SA and Hawkins S and Kenway E	Post-coma disturbance and post-traumatic amnesia as nonlinear predictors of cognitive outcome following severe closed head injury: findings from the Westmead Head Injury Project.	PASAT; SDMT; RAVLT; WAIS (vocabulary subtest)	Cognitive assessments completed 12 months after admission to hospital

Hauger SL and Olafsen K and Schnakers C and Andelic N and Nilsen KB and Helseth E and Funderud I and Andersson S and Schanke AK and L√∏vstad M	Cognitive Event-Related Potentials during the Sub-Acute Phase of Severe Traumatic Brain Injury and Their Relationship to Outcome.	Auditory ERP paradigm	6 months follow-up (post-injury)
Hirota S and Inaji M and Nariai T and Hara M and Tamaki M and Maehara T and Tomita H and Tone O	Correlations between Cognitive Impairments and Employment Status in Patients with Diffuse Axonal Injury.	WAIS-R; WMS-R; TMT A&B; WCST; Word Fluency Test (WFT)	Two assessment timepoints: first early after the injury (1.8 ± 1.4 months) and second later in recovery (18.4 ± 6.5 months)
Houlden DA and Taylor AB and Feinstein A and Midha R and Bethune AJ and Stewart CP and Schwartz ML	Early somatosensory evoked potential grades in comatose traumatic brain injury patients predict cognitive and functional outcome.	SSEP; GOS-E; Rivermead Head Injury Follow-Up Questionnaire; 28-item General Health Questionnaire; Stroop Test; SDMT; PASAT	Cognitive testing at 1 year post-injury. SSEP done at 1,3, and 7 days after TBI.
Jaeger M and Deiana G and Nash S and Bar JY and Cotton F and Dailler F and Fischer C and Rode G and Boisson D and Luaute J	Prognostic factors of long-term outcome in cases of severe traumatic brain injury.	Mnenomic efficiency battery (MEB); TEA battery (Test d'Evaluation de l'Attention); Frontal Assessment Battery at Bedside (FAB); GOS-E; FIM; QOLBI questionnaire	2-4 years after injury
Joseph B and Khalil M and Pandit V and Kulvatunyou N and Zangbar B and O'Keeffe T and Asif A and Tang A and Green DJ and Gries L and Friese RS and Rhee P	Adverse effects of admission blood alcohol on long-term cognitive function in patients with traumatic brain injury.	FIM	2-year retrospective analysis.
Juan E and De Lucia M and Tzovara A and Beaud V and Oddo M and Clarke S and Rossetti AO	Prediction of cognitive outcome based on the progression of auditory discrimination during coma.	"Standardized neuropsychological battery during around 90min"	On average 10 +/- 7 days after awakening
Kiflie A and Alias NA and Abdul-Kareem MM and Mar W and Abdullah J and Naing NN	The prognostic value of early follow-up computerized tomography of the brain in adult traumatic brain injury.	STMS (Short Test of Mental Status); GOS-E	At 6 weeks follow up (after MVA)
Kumar R and Husain M and Gupta RK and Hasan KM and Haris M and Agarwal AK and Pandey CM and Narayana PA	Serial changes in the white matter diffusion tensor imaging metrics in moderate traumatic brain injury and correlation with neuro-cognitive function.	Number connection tests (NCT A and B); Figure connection tests (FCT A and B); WAIS (performance subset)	6 months post-injury.

Kushner DS and Johnson-Greene D	Changes in cognition and continence as predictors of rehabilitation outcomes in individuals with severe traumatic brain injury.	FIM	At admission and discharge to rehab center
Lannoo E and Colardyn F and Jannes C and de Soete G	Course of neuropsychological recovery from moderate-to-severe head injury: a 2-year follow-up.	Neuropsychological battery: Dot cancellation, WAIS DSMT, Stroop, PASAT, visual reaction, binary choice, Complex figure, AVLT, COWAT, Wisconsin card sorting (and more)	At acute hospital discharge, at 6 months and 2 years post-injury
Leblanc J and De Guise E and Feyz M and Lamoureux J	Early prediction of language impairment following traumatic brain injury.	Language testing (by speech-language pathologist):<br>Boston Naming Test;<br>Confrontation Naming Test;<br>BDAE: complex ideational material subset; DTLA: verbal absurdities subtest; Semantic Category Naming (animals);<br>Letter Category Naming-word fluency (first letter)	During patient's acute care. Varied depending on mid, moderate or severe TBI (respectively 10, 16, 21 days post admission from acute care setting).
Leclercq M and Couillet J and Azouvi P and Marlier N and Martin Y and Strypstein E and Rousseaux M	Dual task performance after severe diffuse traumatic brain injury or vascular prefrontal damage.	Random number generation;<br>Visual reaction time	Half of TBI group was examined within 6 months post-injury; the other was seen anytime between 6-30 months post-injury
Lehmann U and Pape HC and Seekamp A and Gobiet W and Zech S and Winny M and Molitoris U and Regel G	Long term results after multiple injuries including severe head injury.	Conclusive thinking (performance test system);<br>Visually bound thinking;<br>Linguistic thinking; Intelligence structure test-analogy; Capability of calculating (intelligence structure test-practical calculation); Recent memory and learning performance (AVLT, Rey); Information processing speed (number connecting test);<br>Visual attnetion (d2 test);<br>Concentration (revision test)	At least two years after injury
Leon-Carrion J and Dominguez-Morales MR and Martin JM	Driving with cognitive deficits: neurorehabilitation and legal measures are needed for driving again after severe traumatic brain injury.	FIM; FAM	Before treatment and upon treatment termination

León-Carrión J, Domínguez-Morales MR, Barroso y Martín JM, & Leon-Dominguez U	Recovery of cognitive function during comprehensive rehabilitation after severe traumatic brain injury.	CRECER Clinical Outcome Scale (CRECERCOS); Performance of cognitive function (long term memory, short-term memory, orientation, calculation, attention, mental control, automation, and planning)	At admission and discharge(from rehab center)
Levin HS and Grossman RG and Rose JE and Teasdale G	Long-term neuropsychological outcome of closed head injury.	BPRS (Brief Psychiatric Rating Scale); WAIS; Selective reminding technique; MAE (multilingual aphasia examination) of Benton	Minimum follow-up period of 6 months. Median duration of follow-up was over a year (post injury)
Levin HS and Meyers CA and Grossman RG and Sarwar M	Ventricular enlargement after closed head injury.	WAIS	At least 6 months after injury
Lim C and Verfaellie M and Schnyer D and Lafleche G and Alexander MP	Recovery, long-term cognitive outcome and quality of life following out-of-hospital cardiac arrest.	NART; RAVLT; BVMT-R; TMT B; WCST; Verbal Fluency; BNT; Peabody Picture Vocabulary; Judgment of Line Orientation; Number location; Visual discrimination; TMT A; Grooved pegboard; Finger tapping	3 months and 12 months post-injury
Lutkenhoff ES and Wright MJ and Shrestha V and Real C and McArthur DL and Buitrago-Blanco M and Vespa PM and Monti MM	The subcortical basis of outcome and cognitive impairment in TBI: A longitudinal cohort study.	GOS-E; SDMT; Stroop Color and Word Test (Golden version); TMT A&B; Short-Delay Free Recall (SDFR); Long-Delay Free Recall (LDFR); Recognition Discriminability (RD); CVLT; Rey 15 Item Test + Recognition; Dot Counting Test	GCS conducted immediately following injury. Other (GOS-E and neurophysiological tests) were only conducted at follow-up.
Martin RM and Wright MJ and Lutkenhoff ES and Ellingson BM and Van Horn JD and Tubi M and Alger JR and McArthur DL and Vespa PM	Traumatic hemorrhagic brain injury: impact of location and resorption on cognitive outcome.	SDMT, oral [O] and written [W] versions; Selective Reminding Test; TMT B	6 months post-injury
Mataro M and Poca MA and Sahuquillo J and Pedraza S and Ariza M and Amoros S and Junqu√© C	Neuropsychological outcome in relation to the traumatic coma data bank classification of computed tomography imaging.	Selective reminding test; Rey-Osterrieth complex figure; TMT A&B; WFT (word fluency test); Grooved pegboard test; SDMT; GOS-E	Neuropsychological testing at 6 months post-injury

Muller SV and von Schweder AJ and Frank B and Dengler R and M√ºnte TF and Johannes S	The effects of proprioceptive stimulation on cognitive processes in patients after traumatic brain injury.	WAIS, RFT (recurring figure test), AVLT, d2 concentration-endurance test, Stroop, 2-back paradigm<br>ERP vibration (choice and passive)	6.4 +/- 3.2 months after injury
Mysiw WJ and Bogner JA and Corrigan JD and Fugate LP and Clinchot DM and Kadyan V	The impact of acute care medications on rehabilitation outcome after traumatic brain injury.	FIM	At admission and discharge (in=px rehab)
Najenson T and Groswasser Z and Mendelson L and Hackett P	Rehabilitation outcome of brain damaged patients after severe head injury.	Raven matrices test; WCST	6 months or more following discharge from hospital
Nakase-Richardson R and McNamee S and Howe LL and Massengale J and Peterson M and Barnett SD and Harris O and McCarthy M and Tran J and Scott S and Cifu DX	Descriptive characteristics and rehabilitation outcomes in active duty military personnel and veterans with disorders of consciousness with combat- and noncombat-related brain injury.	Glasgow Coma Scale; FIM; CRS-R; LCF; Galveston Orientation and Amnesia Test (GOAT); Orientation Log (O-Log)	Admission and discharge (of rehabilitation center)
Nakase-Richardson R and Whyte J and Giacino JT and Pavawalla S and Barnett SD and Yablon SA and Sherer M and Kalmar K and Hammond FM and Greenwald B and Horn LJ and Seel R and McCarthy M and Tran J and Walker WC	Longitudinal outcome of patients with disordered consciousness in the NIDRR TBI Model Systems Programs.	Galveton Orientation and Amnesia Test (GOAT); Orientation log (O-Log); DRS; GCS; FIM; Supervision Rating Scale (SRS)	Subjects and their families were contacted at 1, 2, and 5 years post-injury to assess the subjects' level of functioning, including completion of measures such as FIM, DRS, and SRS.
Niemeier JP and Kreutzer JS and Marwitz JH and Gary KW and Ketchum JM	Efficacy of a brief acute neurobehavioural intervention following traumatic brain injury: a preliminary investigation.	Neurobehavioural Rating Scale-Revised (NRS-R); FIM; Satisfaction with Life Scale (SWLS); MMSE	Pre-intervention measures were obtained. Post-intervention measures were obtained immediately and 3 months following intervention. The 3-month follow-up data was obtained via telephone.
Ørbo M, Aslaksen PM, Larsby K, Norli L, Schäfer C, Tande PM, Vangberg TR, & Anke A	Determinants of cognitive outcome in survivors of out-of-hospital cardiac arrest.	CVLT-II; RCFT (Rey); WMS; D-KEFS (TMT, Stroop, verbal fluency); Grooved pegboard; WASI	3 months after OHCA

Ørbo M, Aslaksen PM, Larsby K, Schäfer C, Tande PM, & Anke A	Alterations in cognitive outcome between 3 and 12 months in survivors of out-of-hospital cardiac arrest.	HADS (Hospital Anxiety and Depression Scale); SF-36 (Short Form Health Survey); CVLT-II; RCFT; D-KEFS; TMT 4 ; Grooved pegboard ; TMT 5 from D-KEFS; WASI;	3 months and 12 months after resuscitation
Ørbo M, Aslaksen PM, Larsby K, Schäfer C, Tande PM, Vangberg TR, & Anke A	Relevance of cognition to health-related quality of life in good-outcome survivors of out-of-hospital cardiac arrest.	Grooved pegboard; TMT-5; CVLT; RCFT; TMT-2; TMT-4; Stroop;	3 months after resuscitation
Ørbo MC, Aslaksen PM, Anke A, Tande PM, & Vangberg TR	Cortical Thickness and Cognitive Performance After Out-of-Hospital Cardiac Arrest.	Grooved pegboard; TMT; D-KEFS; CVLT-II	3 and 12 months after cardiac arrest
Paniak CE and Shore DL and Rourke BP	Recovery of memory after severe closed head injury: dissociations in recovery of memory parameters and predictors of outcome.	SR (selective reminding) procedure<br>Yes-no recognition trial	Within 6 months of regaining consciousness and then again after at least 1 year (range: 367 days to 876 days)
Patel MB and McKenna JW and Alvarez JM and Sugiura A and Jenkins JM and Guillamondegui OD and Pandharipande PP	Decreasing adrenergic or sympathetic hyperactivity after severe traumatic brain injury using propranolol and clonidine (DASH After TBI Study): study protocol for a randomized controlled trial.	Tower Test; Galveston Orientation and Amnesia Test; Numbers Reversed and Verbal Analogies; Tests of Cognitive Abilities; Three Word Recall from Modified Orientation Amnesia Test; Story Retelling Immediate Subtest and Delayed Subtest; TMT A&B; Stroop Test; Word Fluency;<br>BADS	At hospital discharge and at 3 and 12 months (post treatment)
Pietrapiana P and Tamietto M and Torrini G and Mezzanato T and Rago R and Perino C	Role of premorbid factors in predicting safe return to driving after severe TBI.	FIM; FAM; VST (visual search test); WAIS-SDS (symbol-digit subtest)	Time since injury varied from 1 to 16 years
Polanowska KE, Sarzyńska-Długosz IM, Paprot AE, Sikorska S, Seniów JB, Karpiński G, Kowalik R, Opolski G, & Członkowska A	Neuropsychological and neurological sequelae of out-of-hospital cardiac arrest and the estimated need for neurorehabilitation: a prospective pilot study.	ACE-R (Addenbrooke's Cognitive Examination - Revised); TMT A&B; RAVLT; Digit span; Benton Visual Retention Test; Spontaneous speech; WCST; Naming tasks; RCFT	4 assessments: first few days after OHCA, 3 months, 6 months, 12 months post-OHCA
Prigatano GP, Bruna O, Mataro M, Muñoz JM, Fernandez S, & Junque C	Initial disturbances of consciousness and resultant impaired awareness in Spanish	PCRS (patient competency rating scale)	Time of testing ranged from 5 to 53 months post injury, average 24.8 months

	patients with traumatic brain injury.		
Ratcliff JJ and Greenspan AI and Goldstein FC and Stringer AY and Bushnik T and Hammond FM and Novack TA and Whyte J and Wright DW	Gender and traumatic brain injury: do the sexes fare differently?	WAIS-digit span; TMT A&B; SDMT; WMS Logical Memory test; RAVLT; Token test, COWAT; Benton Visual Discrimination test, WAIS Block Design test; WCST; Grooved Pegboard	1 year post injury
Regina Schultza, Robyn L. Tate & Michael Perdices	Neuropsychological recovery during the first 12 months after severe traumatic brain injury: A longitudinal study with monthly assessments	MMSE; Digit span (WAIS-III); SDMT; Verbal fluency; Similarities (SIMS from WAIS-III); Zoo map (BADS); Stroop; HVLT (Hopkins verbal learning test); BVMT; Token test; HPNT (hundred pictures naming test); Judgment line of orientation (JLOT); CFT (Medical College of Georgia)	Started testing as soon as possible after emergence from PTA. Testing every month for 12 months (typically starting at about 3 months post-trauma)
Reider-Groswasser I and Cohen M and Costeff H and Groswasser Z	Late CT findings in brain trauma: relationship to cognitive and behavioral sequelae and to vocational outcome.	"Cognitive status" as measured by multidisciplinary team of psychiatrists, psychologists, neuropsychologists	Measured at discharge from hospital (i.e. "when recovery process showed a plateau" between 3-18 months post-injury). Overall vocational status was evaluated 1 year after discharge
Robertson IH and Manly T and Andrade J and Baddeley BT and Yiend J	Oops!': performance correlates of everyday attentional failures in traumatic brain injured and normal subjects.	SART (sustained attention to response task); Conventional continuous-performance-type test; NART; Cognitive failures questionnaire; PASAT; Other neurocognitive tasks (as comparators to SART)	Between 9-18 months post-injury
Rohling ML and Meyers JE and Millis SR	Neuropsychological impairment following traumatic brain injury: a dose-response analysis.	WAIS, TPT (tactual performance test), CT (category test), WMS (Weshcler Memory Scale), SRT (Buschke-Selective reminding task), Seashore rhythm test, Stroop, TMT A&B, FTT (finger-tapping)	Assessed at 1 and 12 months post injury
Saban KL and Smith BM and Collins EG and Pape TL	Sex differences in perceived life satisfaction and functional status one year after severe traumatic brain injury.	SWLS (Satisfaction with Life Scale);<br>FIM	At 1 year post-injury.

Sabedra AR and Kristan J and Raina K and Holm MB and Callaway CW and Guyette FX and Dezfulian C and Doshi AA and Rittenberger JC	Neurocognitive outcomes following successful resuscitation from cardiac arrest.	CAMCI (Computer assessment of mild cognitive impairment)	"After awakening (defined as following commands)" - (which is) following ICU discharge, pre-hospital discharge
Sahni T and Jain M and Prasad R and Sogani SK and Singh VP	Use of hyperbaric oxygen in traumatic brain injury: retrospective analysis of data of 20 patients treated at a tertiary care centre.	DRS; GCS; LCF	1-6 months post injury, retrospectively assessed
Sandhaug M and Andelic N and Berntsen SA and Seiler S and Mygland A	Self and near relative ratings of functional level one year after traumatic brain injury.	Patient Competency Rating Scale (PCRS)	One year after injury.
Sawchyn JM and Mateer CA and Suffield JB	Awareness, emotional adjustment, and injury severity in postacute brain injury.	PCRS; KAS-R; WAIS or estimate was calculated	"Postacute phase" - retrospective assessment
Schalén W, Sonesson B, Messeter K, Nordström G, & Nordström CH	Clinical outcome and cognitive impairment in patients with severe head injuries treated with barbiturate coma.	Verbal synonym test; Classification of geometrical designs; Paired verbal associates, immediate and delayed recall; BVRT; MFDT - memory for design test; Block Design Test from WAIS-R; TMT A&B	Time between injury and test ranged from 4 months to 60 months
Schielke E, Busch MA, Hildenhagen T, Holtkamp M, Küchler I, Harms L, & Masuhr F	Functional, cognitive and emotional long-term outcome of patients with ischemic stroke requiring mechanical ventilation.	MMSE	At 1 and 2 years after stroke
Schmitter-Edgecombe M and Langill M	Costs of a predictable switch between simple cognitive tasks following severe closed-head injury.	WAIS-R; SDMT; TMT A&B; CVLT; WMS; TAWF; COWAT; WCST-64; Stroop	At least one year after injury
Schmitter-Edgecombe M and Wright MJ	Event-based prospective memory following severe closed-head injury.	Baseline working memory task; Focal-cue prospective memory task; Peripheral-cue prospective memory task; WAIS-R DS, SDMT, TMT A&B, CVLT, WMS-R (logical memory, visual reproduction), COWAT, Stroop, WCST	At least one year post-injury (ranging from 1 year post injury to 27 years post injury)
Schmitz TW and Rowley HA and Kawahara TN and Johnson SC	Neural correlates of self-evaluative accuracy after traumatic brain injury.	PCRS (Patient Competency Rating Scale); DSMT; WAIS	One timepoint, between 8 and 12 weeks post-injury (depending on availability)

Sherer M and Sander AM and Maestas KL and Pastorek NJ and Nick TG and Li J	Accuracy of self-reported length of coma and posttraumatic amnesia in persons with medically verified traumatic brain injury.	TMT A&B; RAVLT; Letter fluency test; WAIS-IV; Symbol Search; Coding, and Letter-Number Sequencing subtest	When deemed testable, average 2.6 months post injury
Sigurdardottir S and Andelic N and Wehling E and Anke A and Skandsen T and Holthe OO and Manskow US and Roe C	Return to work after severe traumatic brain injury: a national study with a one-year follow-up of neurocognitive and behavioural outcomes.	CVLT-II; Rey-Osterrieth Complex Figure Test; D-KEFS (TMT 1-5); CWIT; Verbal Letter Fluency Test;<br>WAIS-III (Letter-Number Sequencing subtest); Hospital Anxiety and Depression Scale (HADS); Behaviour Rating Inventory of Executive Functions-Adult version (BRIEF-A)	One year post-injury.
Sigurdardottir S and Andelic N and Wehling E and Roe C and Anke A and Skandsen T and Holthe OO and Jerstad T and Aslaksen PM and Schanke AK	Neuropsychological functioning in a national cohort of severe traumatic brain injury: demographic and acute injury-related predictors.	CVLT-II; Rey-Osterrieth Complex Figure Test; D-KEFS (TMT 1-5); CWIT; Verbal Letter Fluency Test;<br>WAIS-III (Letter-Number Sequencing subtest); Hospital Anxiety and Depression Scale (HADS);	A standardized telephone interview was administered at 3 months postinjury, and an inperson interview and a neuropsychological evaluation were conducted at 1 year postinjury
Skandsen T and Finnanger TG and Andersson S and Lydersen S and Brunner JF and Vik A	Cognitive impairment 3 months after moderate and severe traumatic brain injury: a prospective follow-up study.	WAIS; Grooved pegboard; TMT 1-5; CWIT; SDMT; CCPT-II; CVMT; TCF; CVLT-II; Digit span (WAIS-III); Tower test	Neuropsychological assessments done 3 months post-injury. GOS-E at 3 months and 12 months post-injury. (98 days±10)
Slavoaca D and Birle C and Stan A and Tatomir A and Popa O and Rosu P and Vulcan AM and Chira D and Livint Popa L and Dina C and Vacaras V and Strilciuc S and Vos P	Prediction of Neurocognitive Outcome after Moderate-Severe Traumatic Brain Injury Using Serum Neuron-Specific Enolase and S100 biomarkers.	ERBI; GOSE; MMSE; Processing Speed Index (PSI); Stroop Test	10 and 90 days post injury.
Smania N and Avesani R and Roncari L and Ianes P and Girardi P and Varalta V and Gambini MG and Fiaschi A and Gandolfi M	Factors predicting functional and cognitive recovery following severe traumatic, anoxic, and cerebrovascular brain damage.	DRS; GOS-E; LCF; FIM	Assessed only at discharge.
Smith-Seemiller L and Lovell MR and Franzen MD and Smith SS and Townsend RN	Neuropsychological function in restrained versus unrestrained motor	TMT B; WCST; COWAT; Logical memory (WMS-R); Visual reproduction (WMS-R)	Unclear how soon assessments were performed following MVC

	vehicle occupants who suffer closed head injury.		
Smith-Seemiller L and Lovell MR and Smith S and Markosian N and Townsend RN	Impact of skull fracture on neuropsychological functioning following closed head injury.	TMT B; WCST; Hopkins Verbal learning test (delayed recall)	Patients completed neuropsych evals at trauma center admission
Songara A and Gupta R and Jain N and Rege S and Masand R	Early Cranioplasty in Patients With Posttraumatic Decompressive Craniectomy and Its Correlation with Changes in Cerebral Perfusion Parameters and Neurocognitive Outcome.	GCS, GOS-E, MMSE	Prior to cranioplasty and 1 week and 1 month after CP.
Spikman JM and Timmerman ME and Milders MV and Veenstra WS and van der Naalt J	Social cognition impairments in relation to general cognitive deficits, injury severity, and prefrontal lesions in traumatic brain injury patients.	TMT A&B; RAVLT; Zoo Map test (BADS); Six Elements test (SET); FEEST (Facial Expressions of Emotion-Stimuli and Tests); Cartoon test; FP test; Emotional Empathy Questionnaire (EEQ)	Time between injury/hospitalization and assessment is not clear
Spikman JM and van der Naalt J	Indices of impaired self-awareness in traumatic brain injury patients with focal frontal lesions and executive deficits: implications for outcome measurement.	Executive route-finding task; Differential Outcome Scale (DOS)	GOS-E, DOS 1 year post-injury. RTW, POS, SIP, executive routine-finding task 2-5 years post-injury.
Stålnacke BM, Saveman BI, & Stenberg M	Long-Term Follow-Up of Disability, Cognitive, and Emotional Impairments after Severe Traumatic Brain Injury.	Barrow Neurological Institute Screen for Higher Cerebral Functions	3 months, 1 year and 7 years post injury.
Steinbusch CVM and van Heugten CM and Rasquin SMC and Verbunt JA and Moulaert VRM	Cognitive impairments and subjective cognitive complaints after survival of cardiac arrest: A prospective longitudinal cohort study.	Cognitive-Log; TMT A&B; Verbal fluency test; Paragraph recall (direct and delayed); Adult memory and information processing battery (AMIPB) task A; CFQ (Cognitive failures questionnaire)	Within 2 weeks of cardiac arrest, at 3 months and 1 year (post cardiac arrest)
Stenberg M, Godbolt AK, Nygren De Boussard C, Levi R, & Stålnacke BM	Cognitive Impairment after Severe Traumatic Brain Injury, Clinical Course and Impact on	GOS-E; LCF; BNIS	3 weeks, 3 months, and 1 year after injury

	Outcome: A Swedish-Icelandic Study.		
Tavano A and Galbiati S and Recla M and Bardoni A and Dominici C and Pastore V and Strazzer S	Cognitive recovery after severe traumatic brain injury in children/adolescents and adults: similar positive outcome but different underlying pathways?	Wechsler Intelligence Scale for Children; WAIS; CPT-II; WCST; Corsi Test; Draw-a-bicycle Test; Benton Line Test; Rey Test (Forms A and B)	6 and 12 months post-TBI
Taylor CM and Aird VH and Tate RL and Lammi MH	Sequence of recovery during the course of emergence from the minimally conscious state.	WNSSP (Western Neuro Sensory Stimulation Profile)	At admission to rehab unit
Temkin NR and Holubkov R and Machamer JE and Winn HR and Dikmen SS	Classification and regression trees (CART) for prediction of function at 1 year following head trauma.	WAIS (verbal IQ and performance IQ); TMT B; Selective reminding test (total recell and 4-hour delayed recall); Halstead impairment index	1 month and 1 year after injury
Terayama Y and Meyer JS and Kawamura J	Cognitive recovery correlates with long-term increases of cerebral perfusion after head injury.	CCSE (Cognitive Capacity Screening Examination)	Mean total follow-up interval of 10 years (post head injury)
Tölli A, Höybye C, Bellander BM, & Borg J	Impact of pituitary dysfunction on cognitive and global outcome after traumatic brain injury and aneurysmal subarachnoid haemorrhage.	BNIS; LCF; GOS-E	3, 6 and 12 months post-injury.
Tölli A, Höybye C, Bellander BM, Johansson F, & Borg J	The effect of time on cognitive impairments after non-traumatic subarachnoid haemorrhage and after traumatic brain injury.	BNIS; LCF; HADS; Barthel Index (BI)	At 3, 6 and 12 months post injury/illness
Trexler LE and Zappala G	Neuropathological determinants of acquired attention disorders in traumatic brain injury.	No standardized set of tests used. Core set of tests used for TBI w/o traumatic aphasia: Boston scanning, Boston version of WMS, Block tapping test, CNR computer tasks, Stroop test	Time since injury varied (average: 88.8 weeks)

Tripodis Y and Alosco ML and Zirogiannis N and Gavett BE and Chaisson C and Martin B and McClean MD and Mez J and Kowall N and Stern RA	The Effect of Traumatic Brain Injury History with Loss of Consciousness on Rate of Cognitive Decline Among Older Adults with Normal Cognition and Alzheimer's Disease Dementia.	TMT A & B; WAIS-R; Animal and Vegetable fluency; Boston Naming Test; MMSE; CDR-SB	For the normal and probable AD cohorts, there was an average of 3.2 ± 1.9 and 1.8 ± 1.1 years of follow-up, respectively. Time since TBI not known.
Ueki Y and Mohri M and Matoba T and Kadokami T and Suwa S and Yagi T and Takahashi H and Tanaka N and Hokama Y and Fukuhara R and Onitsuka K and Tachibana E and Yonemoto N and Nagao K	Prognostic Value of Neurological Status on Hospital Arrival for Short-Term Outcome in Patients With Cardiovascular Shock,,ÄÄ- Sub-analysis of the Japanese Circulation Society Cardiovascular Shock Registry.	CPC (Cerebral performance categories)	At 30 days after hospital arrival
Vilkki J and Ahola K and Holst P and Ohman J and Servo A and Heiskanen O	Prediction of psychosocial recovery after head injury with cognitive tests and neurobehavioral ratings.	Similarities test (and free recall); Block design; Digit span (WAIS); Stroop I & II; Spatial learning by standard sub-goals; Object memory evaluation; Modified Benton Visual Retention test; Fluency (number of words beginning with); Category identification; Sorting; Spatial learning	1-8 months (varied per patient) after head injury
Wagner RH and Cifu DX and Keyser-Marcus L	Functional outcome of individuals with traumatic brain injury and lower extremity deep venous thrombosis.	FIM (including COG-FIM)	At admission and discharge
Weber KT, Guimarães VA, Pontes Neto OM, Leite JP, Takayanagui OM, & Santos-Pontelli TE	Predictors of quality of life after moderate to severe traumatic brain injury.	GCS; MMSE; ERBI	6 months after hospital discharge
Whyte J and Nakase-Richardson R and Hammond FM and McNamee S and Giacino JT and Kalmar K and Greenwald BD and Yablon SA and Horn LJ	Functional outcomes in traumatic disorders of consciousness: 5-year outcomes from the National Institute on Disability and Rehabilitation Research Traumatic Brain Injury Model Systems.	DRS; FIM	At the time of rehabilitation discharge, and via patient or caregiver interview at 1, 2, and 5 years postinjury.
Wiegner S and Donders J	Performance on the Wisconsin Card Sorting Test after traumatic brain injury.	WCST; WAIS-R; TMT A&B	Within one year after injury

Wijnen VJ and Eilander HJ and de Gelder B and van Boxtel GJ	Visual processing during recovery from vegetative state to consciousness: comparing behavioral indices to brain responses.	Western Neuro Sensory Stimulation Profile (WNSSP); GOSE; DRS	9 days after admission to rehabilitation center, then every 2 weeks until discharge. Long term follow-up at 2 years.
Wilde MC and Castriotta RJ and Lai JM and Atanasov S and Masel BE and Kuna ST	Cognitive impairment in patients with traumatic brain injury and obstructive sleep apnea.	Psychomotor Vigilance Test; WMS (digit span); Rey Complex Figure Test; Rey Auditory Verbal Learning Test; Finger Tapping Test	After the second MSLT nap.
Williams JM and Gomes F and Drudge OW and Kessler M	Predicting outcome from closed head injury by early assessment of trauma severity.	WAIS; Halstead-Reitan neuropsychological battery	Interval between injury and time of testing average 44 days (min: 11 days, max: 180 days)
Wilson B and Vizor A and Bryant T	Predicting severity of cognitive impairment after severe head injury.	NART; WAIS; Prose recall (WMS); Associate Learning (WMS); Rivermead Behavioural Memory Test; Rey-Osterreith Complex Figure A; Unusual and Usual Views; WCST; Verbal fluency	Time post-injury varied from 1 month to 84 months (mean: 12 months)
Wong GK and Ngai K and Wong A and Lam SW and Mok VC and Yeung J and Rainer T and Wong R and Poon WS	Long-term cognitive dysfunction in patients with traumatic subarachnoid hemorrhage: prevalence and risk factors.	Chinese MMSE; Hong Kong List Learning Test (HKLLT); Rey Osterrieth Complex Figure; Verbal and visual digit span forward and backward from the Chinese WMS; SDMT; Color Trails Test (CTT); Animal fluency; Modified Boston Naming Test (mBNT); GOS-E; SF-36;<br>Geriatric Depressive Scale (GDC)	3-5 years post-injury
Wong GKC and Ngai K and Poon WS and Zheng VZY and Yu C	Cognitive Outcomes of Patients with Traumatic Bifrontal Contusions.	MMSE; MoCA; Hong Kong List Learning Test (HKLLT); Rey Osterrieth Complex Figure Test Chinese WMS; SDMT; Color Trails Test (CTT); Animal fluency; Modified Boston Naming Test (mBNT)	2 years post-injury

Supplementary Table S2: Total occurrence of cognitive instruments applied to coma survivors

Measure	Total occurence
Addenbrooke's Cognitive Exam (ACE-R)	1
Adult memory and information processing battery (AMIPB)	1
Adult self-report scale (ASR)	1
Attentive matrices test	1
Barrow Neurological Institute Screen for Higher Cerebral Functions (BNIS)	5
Behavioural Assessment of Dysexecutive Syndrome (BADS)	3
Behaviour Rating Inventory of Executive Function - Adults (BRIEF-A) *	2
Benton Facial Recognition Test	1
Benton Judgment of Line Orientation Test (JLOT)	4
Benton Multilingual Aphasia Examination (MAE)	1
Benton Visual Discrimination Test	1
Benton Visual Retention Test	2
Beck Hopelessness Scale (BHS)	1
Boston Diagnostic Aphasia Examination (BDAE)	1
Boston Naming Test	7
Brief Psychiatric Rating Scale (BPRS)	1
Brief Post-Coma Scale (BPCS)	1
Brief Visual Memory Test (BVMT)	5
Buschke Selective Reminding Task	2
Cerebral Performance Categories (CPC)	1
Conners' Continuous Performance Test (CCPT-II)	3
Cognitive Capacity Screening Evaluation (CCSE)	1
Computer Assessment of Mild Cognitive Impairment (CAMCI)	1
Clinical Dementia Rating Scale (CDR)	1
Complex Figures Test (Medical College of Georgia version)	1

Cognitive Failures Questionnaire (CFQ) *	2
Cognitive Log (Cog-Log)	1
CogState	2
Cognitive Test for Delirium (CTD)	1
California Verbal Learning Test (CVLT)	13
Continuous Visual Memory Test (CVMT) *	2
Color Trails Test (CTT) *	2
Corsi Block Tapping Test	2
Controlled Oral Word Association Test (COWAT)	9
Continuous Performance Test	1
Coma Recovery Scale (CRS-R)	4
Confrontation Naming Test	1
CRECER Clinical Outcome Scale (CRECERCOS)	1
Daily Fatigue Impact Scale (DFIS)	1
Delis-Kaplan Executive Functioning System (D-KEFS)	3 full battery; 13 subtests only
Detroit Tests of Learning Aptitude (DTLA)	1
Differential Outcome Scale (DOS)	1
Digit Span	17
Digit Symbol Substitution Test (DSST)	1
Digit Symbol Modality Test (DSMT)	2
Disability Rating Scale (DRS)	12
Dot Cancellation Test	1
Dot Counting Test	1
"Draw-a-bicycle" test	1
D2 Test of Attention	2
Early Rehabilitation Barthel Index (ERBI)	3
Emotional Empathy Questionnaire (EEQ)	1

Executive Route Finding Test (EFRT)	1
Fame Judgment Task	1
Faux Pas Test	1
Frontal Assessment Battery at Bedside (FAB)	1
Facial Expressions of Emotion: Stimuli and Tests (FEEST)	1
Figure Connection Test (FCT)	1
Functional Independence Measure (FIM)	22
Geriatric Depressive Scale (GDS)	1
General Health Questionnaire (GHQ-28)	1
Glasgow Outcome Scale Extended (GOS-E)	16
Grooved Pegboard Test	11
Halstead-Reitan Neuropsychological Battery	4 full battery; 22 subtests only
Hospital Anxiety and Depression Scale (HADS) *	3
Hong Kong List Learning Test (HKLLT)	2
Hopkins Verbal Learning Test (HVLT)	2
Hundred Pictures Naming Test (HPNT)	1
Inglis Paired Associates Test	1
Intelligence Structure Test	2
Katz Adjustment Scale (KAS-R)	2
Lumos Labs NeuroCognitive Performance Test	1
Memory for Design Test (MFDT)	1
Multidimensional health locus of control (MHLC scale)	1
Mill Hill vocabulary scale	1
Mini-Mental State Exam (MMSE)	12
Minnesota Multiphasic Personality Inventory (MMPI)	2
Mnemonic Efficiency Battery (MEB)	1

Montreal Cognitive Assessment (MoCA)	1
National Adult Reading Test (NART)	4
Neurobehavioral Rating Scale - Revised (NRS-R)	1
Number Connection Test	2
Number Location Test	2
Object Memory Test	1
Orientation Log (O-Log)	2
Paced Auditory Serial Addition Test (PASAT)	5
Paired Associates Test	2
Paragraph Recall	1
Patient Competency Rating Scale (PCRS)	4
Peabody Picture Vocabulary	2
Persian Aphasia Test (PAT)	1
Prospective Memory Task	2
Psychomotor Vigilance Test	1
Rancho Los Amigos Levels of Cognitive Functioning (LCF)	16
Random Number Generation	1
Ravens' Progressive Matrices Test	4
Rivermead Behavioural Memory Test (RBMT)	4
Rey Auditory Verbal Learning Test (RAVLT)	13
Rey's Complex Figure Test (RCFT)	19
Rey 15-item Test	1
Recurring Figure Test (RFT)	1
Revised Internal External Scale (RIES)	1
Sustained Attention to Response Task (SART)	1
Screen for Higher Cerebral Functions (BNIS)	1
Selective Reminding Test	6
Sensory Tool to Assess Responsiveness (STAR)	1

Six Elements Test (SET)	1
Short Test of Mental Status (STMS)	1
Spatial Learning	2
Stroop Test	18
Supermarket test	1
Supervision Rating Scale (SRS)	1
Symbol Digit Modalities Test (SDMT)	15
Taylor Complex Figure	1
Test of Adult Word Finding (TAWF)	1
Test d’Evaluation de l’Attention (TEA) battery	1
Telephone Interview for Cognitive Status (TICS)	1
Token Test	2
Trail Making Test (TMT)	46
Unusual Views Test	1
Verbal Fluency Test	18
Visual Object and Space Perception Battery	2
Visual Discrimination	2
Visual Reaction Time	2
Visual Search Test	1
Wechsler Adult Intelligence Scale (WAIS)	23 full battery; 30 subtests only
Wechsler Abbreviated Scale of Intelligence (WASI)	3
Wechsler Memory Scale (WMS)	11 full battery; 14 subtests only
Wessex Head Injury Matrix (WHIM)	1
Western Neuro Sensory Stimulation Profile (WNSSP)	2
Wisconsin Card Sorting Test (WCST)	19
Wisconsin General Test Apparatus (WGAT) - adapted for humans	3

Supplementary Table S3: Description and Method of Administration of Cognitive Instruments used in Three or More Articles

Measure	Subscale (if applicable)	Description	Administration	Freq.
Barrow Neurological Institute Screen for Higher Cerebral Functions (BNIS)		38-item test which examines 7 functions: language, orientation, concentration, visuospatial function, memory, affect, and self-evaluation of performance	Total score is sum of 7 sub-scores. Does not require training for clinicians	5
Behavioural Assessment of Dysexecutive Syndrome (BADS)	Zoo Map Test	Participants are given a map of a zoo and instructions of places they have to visit. Test of planning abilities (formulation and execution)	Scoring based on total number of errors and times taken to plan and execute the routes.	3
Benton Judgment of Line Orientation Test (JLOT)		Measures visuospatial judgment in brain-injured patients. The test examines the ability of a subject to estimate angular relationships between line segments by visually matching angled line pairs to 11 numbered radii forming a semi-circle.	Subjects are given a stimulus booklet and asked to match two angled lines to a set of 11 lines that are arranged in a semicircle and separated 18 degrees from each other. The complete test has 30 items.	4
Boston Naming Test		Neuropsychological assessment tool to measure confrontational word retrieval in individuals with aphasia or other language disturbance caused by stroke, Alzheimer's disease, etc.	60-item test, it aims to determine the extent of the subject's visual confrontation naming abilities. A 15-item short form and a multiple-choice version of the test can be used to retest comprehension of any words missed on the confrontation naming exercise.	7
Brief Visual Memory Test (BVMT)		Measure of visuospatial memory and memory abilities. Subjects are asked to look at six stimuli (geometric figures) and subsequently draw as many as possible in their correct location. A Delayed Recall Trial is administered after a 25-minute delay. Lastly, a Recognition Trial, in which the subject is asked to identify which of 12 figures were included among the original geometric figures.	Total administration time is 45 min approx.	5

Conners' Continuous Performance Test (CCPT-II)		Standardized, computerized instrument designed to assess different aspects of attention/executive functions.	Consists of 360 single letters presented one by one on a computer screen in 18 consecutive blocks of 20 trials. Respondents are asked to press a button every time a stimulus (letter) is presented, except for the letter "X." It takes 12 minutes to complete.	3
California Verbal Learning Test (CVLT)		Measures episodic verbal learning and memory, and demonstrates sensitivity to a range of clinical conditions.	The experimenter reads a list of 16 nouns aloud, at one-second intervals, in fixed order, over five learning trials. After each trial, the subject is asked to recall as many words as they can in any order (i.e., free recall). An interference list of words is used too and the test ends with a word recognition task. Total administration time is around 30 min.	13
Controlled Oral Word Association Test (COWAT)		Verbal fluency tasks evaluate the spontaneous production of words under restricted search conditions.	This test requires the individual to name as many words as possible that begin with a given letter. Sixty seconds are allotted for each letter. Administration takes around 5 minutes.	9
Coma Recovery Scale (CRS-R)		Used to assist with differential diagnosis, prognostic assessment, and treatment planning in patients with disorders of consciousness. It consists of 23 items that comprise six subscales addressing auditory, visual, motor, oromotor, communication and arousal functions.	Time to administer between 15 and 30 min.	4
Delis-Kaplan Executive Functioning System (D-KEFS)	*Whole battery	Standardized assessment of executive functions including flexibility of thinking, inhibition, problem solving, planning, impulse control, concept formation, abstract thinking, and creativity. It is composed of nine stand-alone tests that can be individually or group administered.	Administration of the full battery takes 90 minutes.	3

	Verbal fluency	Assess verbal behavioural productivity and cognitive flexibility.	Composed of three testing conditions: Letter Fluency, Category Fluency, and Category Switching.	5
	Color-Word Interference Test (CWIT)	Measures multiple aspects of visual behavioral productivity and cognitive flexibility. DF evaluates visual productivity with and without distracting stimuli, and switching between stimuli		4
	Tower Test	Measures planning and reasoning in the spatial modality, impulsivity.		4
Digit Span				17
Disability Rating Scale (DRS)		This scale was developed as a measure of disability that would be applicable to individuals with moderate to severe traumatic brain injury at a wide range of functional levels from coma through community living. It has eight areas of functioning components: Eye Opening, Best Communication Ability, Best Motor Response, Cognitive Ability (Feeding, Toileting and Grooming, Level of Functioning and Employability.	The scale can be self-administered or scored through an interview with the participant or a family member.	12
Early Rehabilitation Barthel Index (ERBI)	-aka Barthel index	Ordinal scale used to measure performance in ADLs. Ten variables describing ADLs are scored. The Barthel Index measures the degree of assistance required by an individual for mobility and self-care.	Self-report (5 minutes) & direct observation (20 minutes)	3
Functional Independence Measure (FIM)	Motor and Cognitive subtest	Developed as a measure of disability for a variety of populations. 18-item 7-level ordinal scale intended to be sensitive to changes over the course of a comprehensive inpatient medical rehab program.<br>Cognition subscale includes: comprehension, expression, social interaction, problem solving, memory	Each item is scored on a 7 point ordinal scale (by a rater).<br>Takes approximately 30-45 minutes to administer.	22

Glasgow Outcome Scale Extended (GOS-E)		A test to classify global outcomes in TBI survivors. It is a “global scale for functional outcome that rates patient status into one of five categories: dead, vegetative, severe disability, moderate disability, and good recovery”	Use of the structured interview is recommended to facilitate consistency in ratings since this test isp rated by an examiner.	16
Grooved Pegboard Test		Manipulative dexterity test that contains 25 holes with randomly positioned slots and pegs which have a key along one side. Pegs must be rotated to match the hole before they can be inserted.	Measures performance speed in a fine motor task.	11
Halstead-Reitan Neuropsychological Battery	Whole battery	The full HRNB includes: Weschler Intelligence Scale, Aphasia Screening Test, Trail-Making Test, Halstead Category Test, Tactual Performance Test, Seashore Rhythm Test, Speech Sounds Perception Test, Finger Tapping Test, Sensory Perceptual Examination, Lateral Dominance Examination	Used to evaluate the “condition and functioning of the brain, including etiology, type, localization and lateralization of brain injury.”<br><br>Administration of the full battery takes over 3 hours.	4
	Finger Tapping/Oscillation Test	Participant places hand on flat surface and uses 2 fingers on the same hand to tap two buttons, alternating.	Test that examines motor functioning (motor speed and lateralized coordination).	6
	Speech Sounds Perception Test	Measures a person’s ability to match a spoken nonsense word containing double e (e.g. teeg) with its written counterpart		2
	Seashore Rhythm Test	Participant listens to a recording of pairs of rhythmic patterns and indicates whether they are the same or different.		3
	Tactual Performance Test	Participants are blindfolded and are asked to place wooden shapes into a formboard placed at a 45 degree angle.	Measures motor abilities and recall of motor stimuli, as well as nonverbal memory and other cognitive functions.	4
	Category Test	Participant is presented with 6 subtests - each has a different set of stimuli organized according to a specific principle, and each stimulus is associated with a particular number. Participants must	Non-verbal problem-solving task that requires abstract reasoning, concept formation, and mental flexibility.	7

		choose a number and use feedback/response accuracy to solve each item.		
Mini-Mental State Exam (MMSE)	-	Scale used to measure cognitive impairment in older adults. It assesses different subsets of cognitive status including attention, language, memory, orientation, visuospatial proficiency.	Objective questions administered by a rater (10-15 minutes)	12
National Adult Reading Test (NART)		The test comprises 50 written words in English which all have irregular spellings, so as to test the participant's vocabulary rather than ability to apply pronunciation rules.	Used to estimate pre-morbid intelligence, because it assesses abilities that are thought to be spared following neurological injury.	4
Paced Auditory Serial Addition Test (PASAT)		Participants are given a number every 3 seconds and are asked to add the number they just heard to the number they heard before.	Used to assess capacity and rate of information processing and sustained and divided attention. This task involves working memory, attention, and arithmetic capabilities.	5
Patient Competency Rating Scale (PCRS)		Scale that aims to assess self-awareness following brain injury. Consists of 30 items that specify different tasks and functions in four domains (ADLs, behavioural and emotional function, cognitive abilities, and physical function).	Participant responds on a 5-point scale to each of the 30 items. An informant rating should be obtained from a relative or therapist.	4
Rancho Los Amigos Levels of Cognitive Functioning (LCF)		Describes the cognitive and behavioural patterns found in brain injury patients as they recover from coma.<br>The scale consists of 8 levels. Each level is presented on the scale as a narrative form and the rater must decide which level best describes the patient's present behaviours.	The scale was based on the assumption that observation of the type, nature, and quality of a patient's behavioural responses can be used to estimate the cognitive level at which the patient is functioning.	16
Ravens' Progressive Matrices Test		Nonverbal test used to measure human intelligence. In each test item, subject is asked to identify the missing element that completes a pattern.	60 multiple choice questions. Measures reasoning ability.	4
Rivermead Behavioural Memory Test (RBMT)	14 subtests	Subtests include:<br>First and second names (delayed recall): asked to remember names of people in photos	Assesses aspects of visual, verbal, recall, recognition, immediate and delayed everyday	4

		Belongings (delayed recall): asked to remember what objects were hidden at the beginning of the test<br>Appointments (delayed recall): when an alarm rings, participant has to answer specific questions<br>Story (immediate & delayed)<br>Picture recognition (delayed)<br>Face recognition (delayed)<br>Route (immediate and delayed): same as zoo map test<br>Messages (immediate and delayed)<br>Orientation<br>Novel task (immediate and delayed): use different coloured pieces to make a shape as demonstrated by examiner	memory, prospective memory skills, ability to learn. It takes approx 30 minutes to complete.	
Rey Auditory Verbal Learning Test (RAVLT)		Five presentations of a 15-word list are given, each followed by attempted recall. This is followed by a 15-word interference list (list B), followed by recall of list A.<br>This test is succeeded by CVLT.	Evaluates short-term memory, rate of learning, interference, and other executive functions. Approximately 10-15 minutes required (plus 30 minute interval)	13
Rey's Complex Figure Test (RCFT)		Participants are asked to reproduce a complicated line drawing, first by copying it freehand and then drawing from memory.	Captures information about five cognitive domains: visuospatial recall memory, visuospatial recognition memory, response bias, processing speed, visuospatial constructional ability.	19
Selective Reminding Test		Test designed to measure verbal learning and memory through use of a list-learning procedure over multiple trials (participant is asked to remember a list of words; on next trial, participant is only reminded of missed words from previous trial). This test distinguishes short-term memory from long-term storage	Approximately 30 minutes.	6
Stroop Test	-	Test used to assess the ability to inhibit cognitive interference.	Objective measure, scored on speed and accuracy	18

Symbol Digit Modalities Test (SDMT)	-	Simple substitution task (where the examinee pairs specific numbers with geometric figures), sensitive to cognitive changes over time.<br>*DSMT=DSST=SDMT	Scored on speed and accuracy (90 second task)	15
Trail Making Test (TMT)	Subtests A and B	Test in which participants must link numbers from 1-25 or numbers (1-12) and letters (A-L) in order.	Timed assessment. Reviewer must be present to correct if the participant makes a mistake.	46
Verbal Fluency Test	Animal, Vegetable, etc.	Short test of verbal functioning. Typically 2 tasks (category/semantic fluency and letter/phonemic fluency)	Scored on number of unique words	18
Wechsler Adult Intelligence Scale (WAIS)	*Full battery	IQ test, designed to measure intelligence and cognitive ability.<br>Full scale IQ = Verbal IQ (verbal comprehension index + working memory index) + Performance IQ (perceptual organization index + processing speed index)		23 (+3)
	Block design	Test of perceptual organization index (within performance IQ subindex)		6
	Processing Speed Index (PSI)	Measures the speed of mental processing, using visual stimuli and graphomotor skills. Includes symbol search and digit symbol coding tasks. These tasks require attending to visual material, visual perception and organization, visual scanning, and hand-eye coordination.	Timed assessments	4
	Digit Span	Test of working memory within verbal IQ index		5
	Symbol Search	Test of processing speed within performance IQ index		1
	Digit Symbol Modalities Test (DSMT)	Test of processing speed within performance IQ index		3

	Similarities	Test of verbal comprehension within verbal IQ index		4
	Vocabulary	Test of verbal comprehension within verbal IQ index		2
	Letter-Number sequencing (LN)	Test of working memory within verbal IQ index		2
Wechsler Abbreviated Scale of Intelligence (WASI) *		Short form of the WAIS used to estimate intellectual functioning. 4 subtests: 2 verbal, 2 performance	Approximately 20 minutes in length	3
Wechsler Memory Scale (WMS)	*Full battery	Designed to be used WITH the WAIS.<br>• WMS-IV is made up of seven subtests: Spatial Addition, Symbol Span, Design Memory, General Cognitive Screener, Logical Memory (I & II), Verbal Paired Associates (I & II), and Visual Reproduction (I & II)	A person's performance is reported as 5 index scores: auditory memory, visual memory, visual working memory, immediate memory, and delayed memory	11
	Digit Span	*has since been eliminated from WMS. Measures verbal STM		2
	Spatial Addition	Evaluates visual-spatial working memory using a visual addition task. The participant is shown two images with red and blue circles, and asked to add or subtract the locations of the circles.		-
	Design Memory	Assesses memory for visual images in a grid (requiring memory of both visual and spatial information)		-
	Symbol Span	Assesses visual working memory. Uses novel visual stimuli (difficult to verbalize) and requires storage and manipulation functions.		-
	Logical Memory (I&II)	Participant is read a short prose passage and asked to recall as many details as possible both immediately and after delay.	Takes approximately 5 minutes to administer, but requires time for delay.	8

	Verbal Paired Associates (I&II)	Required to recall novel and semantically related word pairs after 20-30 minute delay. Measure of delayed cued recall for word associations		-
	Visual Reproduction (I&II)	Test of nonverbal delayed recall.		4
Wisconsin Card Sorting Test (WCST)		Stimulus cards are presented to the participant, who must match the cards (but are not told how). Test of “set-shifting” (ability to display flexibility in changing schedules of reinforcement)	Takes ~12-20 minutes to carry out. Generates scores of categories achieved, trials, errors, and perseverative errors.	19
